# Spatial Applicability of Fictitious Point Feedback for Frequency Response Prediction in Unidirectional Carbon Fiber-Reinforced Polymer Plates

**DOI:** 10.3390/polym18212631

**Published:** 2026-10-23

**Authors:** Chan-Jung Kim

**Affiliations:** School of Mechanical Engineering, Pukyong National University, 45, Yongso-ro, Nam-gu, Busan 48513, Republic of Korea; cjkim@pknu.ac.kr

**Keywords:** carbon fiber-reinforced polymer, fiber orientation, frequency response function, fictitious feedback, laser vibrometry, spatial correlation, anisotropy

## Abstract

Fiber orientation and anisotropy change the spatial vibration response of carbon fiber-reinforced polymer (CFRP) plates, potentially limiting prediction from a single driving-point frequency response function (FRF). This retrospective study assesses fictitious feedback using complex mobility FRFs measured by laser vibrometry at 15 locations for seven CFRP orientations and a thicker matrix-only specimen. One specimen and one averaged FRF dataset were analyzed per configuration. A frequency-dependent scalar was calibrated at P8, with 14 outputs reserved for validation. All 42 directed CFRP comparisons were evaluated using complex error, spatial correlation, and frequency coverage. Within 100–1000 Hz, 28 comparisons exceeded 100% error. The lowest error, 18.2% for 90–75°, covered 36.8% of frequency lines and 26.9% of validation cells, and 96.8% of retained squared target-response amplitude lay within 200–300 Hz. The same comparison gave 45.3% without rescaling and 40.2% with a constant P8-fitted scalar, but local errors exceeded 90%. The frequency-dependent scalar outperformed the constant scalar in only 14 of 42 primary-band comparisons. These observations define conditional scalar-transfer limitations for the tested specimens and support arrangement. They do not establish prospective accuracy at unmeasured outputs or isolate fiber orientation from specimen and support effects.

## 1. Introduction

Carbon fiber-reinforced polymer (CFRP) is used in lightweight load-bearing structures because of its high specific stiffness and strength and the ability to tailor properties through fiber orientation. Fatigue resistance and environmental durability depend on laminate architecture, matrix properties, and exposure rather than being universal advantages over every other composite. Guo et al. reviewed CFRP fatigue mechanisms [1]. Xian et al. studied prestressed CFRP plates under hygrothermal and freeze–thaw exposure [2], while Yu et al. examined carbon–glass hybrid bars in water and alkaline solution [3]. These studies motivate careful material and processing description; they do not supply constitutive parameters for the present UD175/M.008 plates.

Classical lamination theory describes the directional stiffness of unidirectional CFRP laminates by transforming lamina properties into structural coordinates and assembling the extensional, coupling, and bending stiffness matrices [4]. Changes in resonance frequency with fiber orientation are therefore expected consequences of anisotropic mechanics. A distinct experimental question is whether a spatial frequency response function (FRF) dataset from one specimen, supplemented by a single driving-point measurement on another, can support response prediction across fiber orientations without constructing a complete structural model.

Composite damping also depends on fiber orientation. Even when polymer dissipation contributes substantially, the energy dissipated by a vibration mode depends on its strain distribution and the directional complex properties of the laminate. Strain-energy and complex-stiffness formulations account for these effects [5,6]. Unchanged matrix chemistry does not imply orientation-independent modal damping, and an inverse FRF is not an independently measured material damping coefficient. These considerations motivate an assessment of measured complex responses without attributing their changes exclusively to stiffness or damping.

Earlier studies tracked corresponding CFRP modes using resonance frequencies, damping information, and modal vectors [7], estimated system parameters from reference-angle modal information [8], and introduced spatial weighting of mode-shape vectors to improve modal correspondence [9]. Off-axis reinforcement changes the measured spatial response and complicates conventional bending or torsional classification. These methods therefore depend on identifiable modal quantities or calibrated relationships between orientations.

Recent research has pursued complementary approaches to composite dynamics. Milewski [10] combined experiments and finite element analysis to examine how small ply-angle deviations affect CFRP bending and modal responses. Hedayatrasa et al. [11] developed a k-space physics-informed neural network to reconstruct composite-laminate bending mode shapes and identify effective bending stiffness from noisy, finite-element-generated data. Zanetti et al. [12] evaluated delamination using amplitude- and phase-based FRF indices for different CFRP laminate configurations. These studies address orientation sensitivity, reconstruction, and damage assessment, respectively. The present question concerns the spatial transferability of a single complex scaling factor between experimentally measured specimens.

Frequency-domain modal estimation methods, including PolyMAX, provide systematic procedures for extracting poles and modal vectors [13]. Their usable information depends on excitation, spatial sampling, signal quality, and resonance separation. Comparing complex forced-response vectors directly permits a feasibility assessment before assigning individual mode labels. Laser vibrometry avoids the mass of an attached response accelerometer [14]. Recent experiments on lightweight CFRP structures also show the importance of support conditions and sensor mass loading [15]. Noncontact sensing addresses the latter, but does not eliminate residual support effects or excitation limitations.

Receptance-based structural modification and feedback provide an established framework for response prediction [16]. Fictitious proportional feedback has been used for frequency-domain identification of an active magnetic bearing rotor system [17], and a recent review connects structural modification with active vibration control through receptance identities [18]. Shi and Li [19] extended the approach to higher-rank modifications for sound-power control at multiple frequencies, demonstrating their method with numerical examples. Such developments emphasize the importance of the spatial form of the modification. Here, the modification is restricted to a fictitious scalar feedback at the measured central driving point. A frequency-dependent complex gain can reproduce the target driving-point FRF algebraically when both driving-point measurements are available. Agreement there cannot establish whether the same gain predicts responses elsewhere or represents distributed anisotropic reinforcement. The unresolved practical issue is the extent to which this restricted modification transfers across the measured response field, and how to distinguish inadequate driving-point calibration from an irreducible mismatch between spatial response patterns.

The unresolved question is how accurately a scalar calibrated from one driving-point FRF transfers across measured spatial responses after fiber reorientation. We assess this question retrospectively using all 42 directed comparisons among seven CFRP specimens, a separate matrix-only comparison, and matched-mask benchmarks against unadjusted and constant-scalar predictions. The scalar feedback algebra, least-squares projection, and response vector assurance criterion (RVAC) are established tools [16,17,18,19,20]. The contribution is experimental evidence of conditional spatial transfer and its failure: error distributions, excluded observations, local residuals, and amplitude weights delimit the observed domain. The 42 comparisons reuse seven specimens and are not independent experiments. Reference selection is exploratory rather than an independently validated optimization. Target validation responses and their coherence define the assessment domain and retrospective diagnostics; they are not amplitude inputs to the P8-ratio predictor. The feedback representation expresses an equivalent single-coordinate dynamic-stiffness change without identifying distributed laminate reinforcement or a realizable controller.

## 2. Materials and Methods

### 2.1. Specimens and Coordinate Convention

The dataset comprised seven unidirectional (UD) CFRP specimens with fiber orientations of 0°, 15°, 30°, 45°, 60°, 75°, and 90°, together with one matrix-only specimen. The measured CFRP widths, lengths, and thicknesses were within 79.9–80.1 mm, 149.9–150.1 mm, and 1.9–2.1 mm, respectively; masses were 37.3–37.5 g. These are observed measurement ranges, not standard deviations or instrument uncertainties. The matrix-only specimen had width and length within the same ranges, a measured thickness of 3.0 mm, and a mass of 44.3 g. It was therefore not a geometry-matched control. Multiple coupons were manufactured, but one tested specimen and one averaged FRF dataset were analyzed per configuration. Independently tested specimen replicates and individual impact records were unavailable for statistical uncertainty analysis. Table 1 summarizes the measured specimen dimensions and masses.

**Figure A1:**
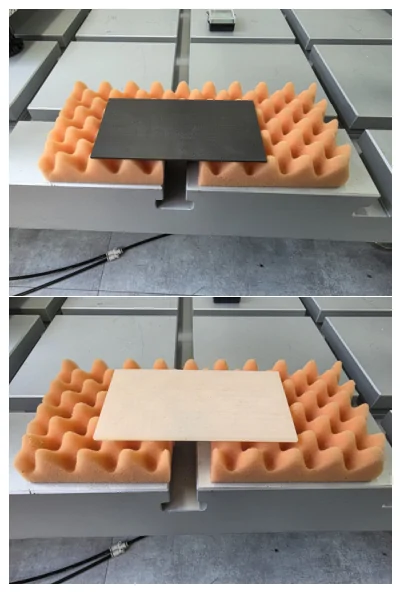
Representative specimen photographs: CFRP (top) and matrix-only plate (bottom). Both plates rest on compliant foam above a rigid surface plate, as used to approximate free–free conditions in the impact tests. The fiber angle of the photographed CFRP plate is not assigned from its surface appearance.

A nominally 2000 mm × 2000 mm CFRP parent panel of nominal thickness 2 mm was fabricated by stacking 12 UD175 prepreg plies with a common fiber direction. The prepreg was supplied by TB Carbon (Yangsan, Republic of Korea) and used resin system M.008. Its nominal fiber and resin areal masses were 175 and 86 g/m^2^, respectively, corresponding to 261 g/m^2^ total areal mass and 33.0% resin mass fraction. These supplier values were not treated as measurements of cured fiber volume fraction. The nominal cured ply thickness was 0.169 mm. The parent panel was autoclave-cured at 130 °C for 90 min, and coupons were cut at the seven orientations relative to the specimen axes. Cutting from one parent panel reduced differences associated with separate layup and curing batches, but did not quantify within-panel variability. A separate large matrix-only panel was produced from the same M.008 resin using the same autoclave curing procedure and cut into coupons. Its measured thickness was 3.0 mm. Figure A1 shows representative specimens.

**Figure 1:**
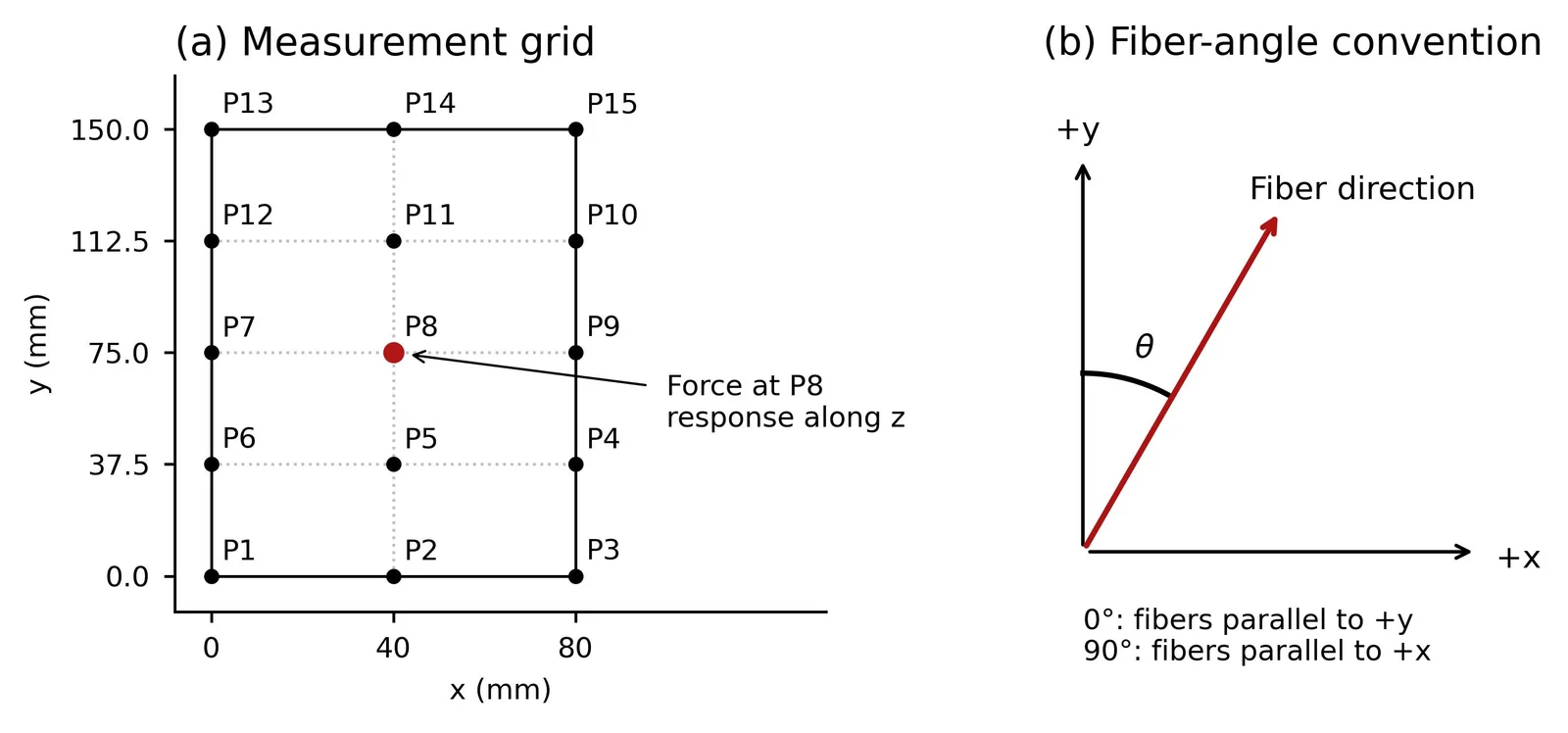
Measurement coordinates and fiber-angle convention: (**a**) 15-point grid and central force at P8; (**b**) fiber direction measured clockwise from +y toward +x when viewed from +z. The response direction is z. Table A1 lists the coordinates. Support hardware is described in Section 2.2.

The width, length, and out-of-plane coordinates were denoted by x, y, and z, respectively. The 0° fiber direction was parallel to the y-axis. The positive fiber angle θ was measured clockwise from +y toward +x when viewed from +z; thus, 90° corresponded to the x-direction. The in-plane unit vector along the fibers was $d_{f}\left(\theta \right)=\left(sin\theta ,cos\theta \right)$, where the subscript f identifies the fiber direction. The conventional lamina angle measured counterclockwise from +x was $\alpha =90^{\circ }-\theta$. This relation was used to connect the specimen orientation labels to the transformed stiffness coefficients. The measurement coordinates and fiber-angle convention are shown in Figure 1.

**Figure A2:**
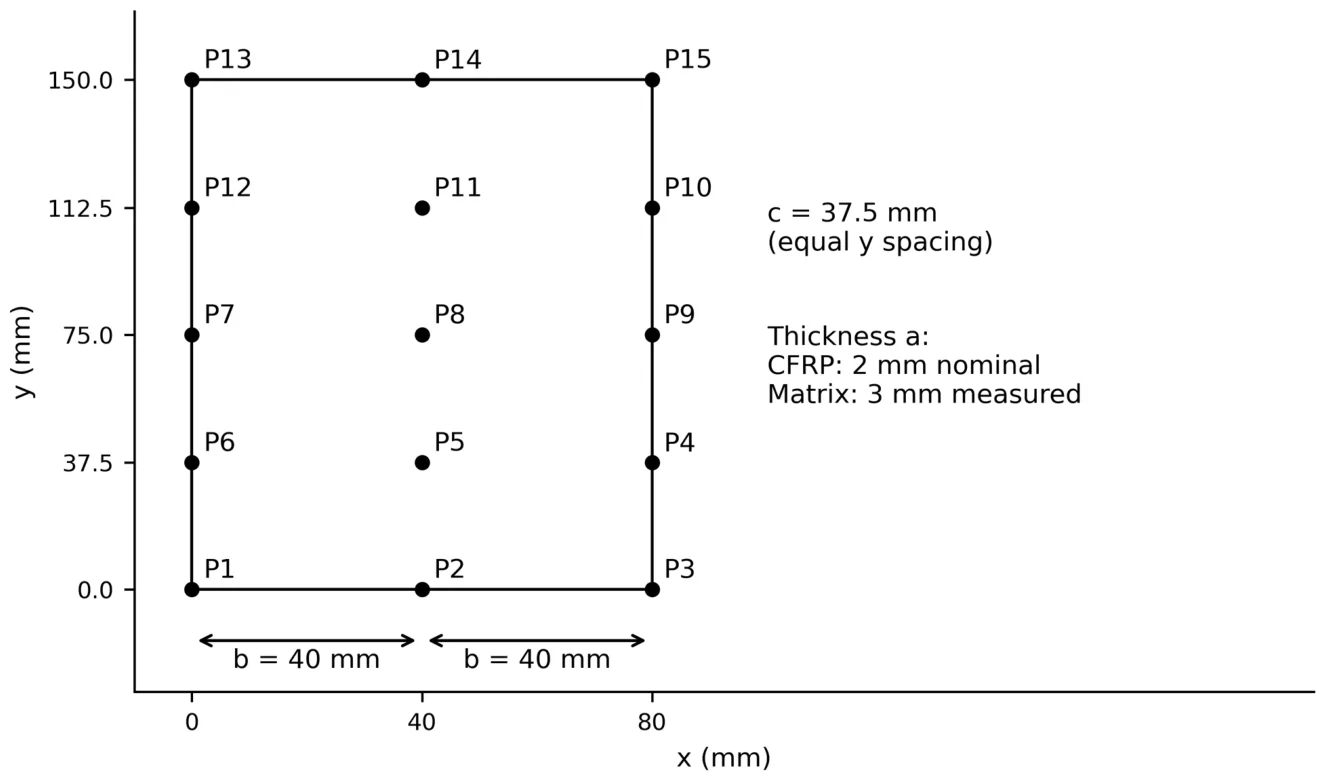
Plan dimensions and response grid. Nominal spacings are b = 40 mm and c = 37.5 mm. Nominal CFRP thickness is 2 mm (measured range 1.9–2.1 mm); matrix thickness is 3.0 mm. Both configurations have nominal plan dimensions 80 mm × 150 mm. P8 is the physical force location and z is the response direction.

Fifteen response locations formed a 3 × 5 grid with nominal spacings of 40 mm along x and 37.5 mm along y. Figure A2 defines the plan dimensions and distinguishes the nominal 2 mm CFRP thickness from the measured 3 mm matrix thickness. The force was applied at P8 = (40, 75) mm. Figure 1 and Table A1 give the alternating row numbering. Acquisition reference labels were interpreted as channel identifiers rather than physical point numbers; the reference label in the exports did not indicate excitation at P1.

### 2.2. Experimental Measurements and Data Preparation

Each specimen was placed on laboratory foam larger than its plan dimensions. The foam rested on a high-stiffness SUS304 surface plate supported by four air-suspension units. The same arrangement was used for all configurations. The foam material specification, stiffness, damping, and contact-pressure distribution were not characterized, and no measurements with altered support or suspension were performed. Air suspension was intended to reduce transmission of floor vibration to the surface plate but did not demonstrate negligible foam–specimen interaction. The boundary condition is therefore described as nominally free–free. Out-of-plane excitation was applied at P8 using an instrumented impact hammer (5800B3, Dytran, Chatsworth, CA, USA) fitted with a steel tip. During preliminary Test.Lab bandwidth setup, the force spectrum was checked to confirm a roll-off of less than 10 dB over the intended excitation band. Out-of-plane responses were measured at P1–P15 with a PSV-500-A laser vibrometer (Polytec GmbH, Waldbronn, Germany), comprising a PSV-I-500 scanning head and PSV-F-500 front-end unit. The single-input multiple-output (SIMO) data used one physical force location and velocity-to-force FRFs, denoted by Y, in (m/s)/N. Laser measurement avoided an attached response-sensor mass.

Acquisition used the Impact Testing module of Test.Lab 15A (Siemens). At each response point, ten impact measurements were used to obtain the exported averaged FRF. The input Force-Exponential window used the software-suggested cutoff setting of 1.12%. The response Exponential window used a decay setting of 100%, equivalent to uniform weighting without exponential attenuation [21]. Accordingly, response exponential weighting did not add artificial damping. This does not eliminate possible finite-record leakage or input-window effects. Each configuration contained 15 complex FRFs and 15 magnitude-squared coherence traces on 25,600 frequency lines from 0.3125 to 8000 Hz, with a spacing of 0.3125 Hz. Real and imaginary components were retained. FRFs and coherence had different channel orders and were aligned using response-point identifiers. Checks confirmed finite entries, a common frequency grid, and coherence values within [0, 1]. Ten-impact averaging was distinguished from independent specimen replication; the individual impact records were not available.

The archived text headers explicitly report a sample frequency of 16,000.6 Hz and frequency spacing of 0.3125 Hz. They also report a Span of 6250.24 Hz, whereas the numerical frequency arrays extend to 8000 Hz. The original acquisition project was not supplied, so this metadata discrepancy and the validated acquisition passband cannot be resolved from the exports. Analyses above 3000 Hz are therefore descriptive checks of the archived numerical samples. The FRF estimator, detailed spectral averaging settings, trigger rejection settings, record duration, and any sign or calibration conversion beyond the exported +Z labels and mobility units are not independently recoverable from these headers. The 15 transfer paths are assembled as one input column; SIMO describes this data arrangement and does not assert simultaneous acquisition. Manufacturing, dimensions, mass ranges, hammer tip, and window settings are reported from the author’s measurement account; contemporaneous per-specimen sheets and acquisition settings files were not included in the retained data.

The original primary and secondary evaluation bands, 100–1000 Hz and 1000 < f ≤ 3000 Hz, were retained for comparability with the submitted assessment. The primary band evaluates a lower-frequency response range; the secondary band tests whether that approximation extends to higher frequencies. The boundaries are analysis choices, not identified modal boundaries or an independently demonstrated support cutoff. To assess truncation of the recorded range, additional results for 3000 < f ≤ 5000 Hz and 5000 < f ≤ 8000 Hz are reported in Section 3.7 and the Supplementary Materials. Frequencies below 100 Hz were excluded to avoid making claims about rigid-body and support-dominated motion, but this exclusion does not establish negligible support effects above 100 Hz. Frequency lines are samples of one averaged experiment, not independent repetitions. The predictor was neither smoothed nor interpolated across frequency.

### 2.3. Fictitious Feedback and Driving-Point Calibration

For a linear reference structure undergoing harmonic motion, the dynamic stiffness and receptance matrices are related by Equation (1).

(1) $$Z_{r}\left(\omega \right)=K_{r}-\omega^{2}M_{r}+i\omega C_{r},\;\;G_{r}=Z_{r}^{-1}.$$

Here, $Z_{r}$ is the reference dynamic stiffness matrix; $K_{r}$, $M_{r}$, and $C_{r}$ are its stiffness, mass, and viscous damping matrices, respectively; and $G_{r}$ is its receptance matrix, which maps harmonic forces to displacements. The subscript r denotes the reference specimen, and the superscript −1 denotes a matrix inverse. The angular frequency is ω=2πf, where f is the frequency in hertz, and $i=\sqrt{-1}$ is the imaginary unit. The harmonic convention $exp\left(i\omega t\right)$ is used throughout, with t denoting time.

Equation (1) provides a theoretical representation of the structure; these matrices were not identified from the SIMO measurements. A frequency-dependent constitutive model can also be incorporated into the dynamic stiffness without changing the subsequent algebra. Fictitious negative displacement feedback at P8 produces the rank-one modification in Equation (2).

(2) $${\hat{Z}}_{t}=Z_{r}+\Gamma \left(\omega \right)bb^{T}.$$

Here, ${\hat{Z}}_{t}$ is the dynamic stiffness matrix predicted for the target specimen, and $Z_{r}$ is the reference dynamic stiffness matrix. A hat denotes a model prediction, and the subscript t denotes the target specimen rather than time. The complex scalar $\Gamma \left(\omega \right)$ is the equivalent displacement-feedback gain at angular frequency ω, with units of N/m. The dimensionless column vector b selects the out-of-plane degree of freedom at P8: its corresponding entry is one, and all other entries are zero. The superscript T denotes the ordinary transpose, so $bb^{T}$ confines the modification to this coordinate. The plus sign follows the negative-feedback sign convention, with the feedback force defined as the negative of the gain multiplied by the driving-point displacement.

Applying the rank-one matrix inverse identity gives the modified receptance column in Equation (3), provided that the reference matrix is invertible and the scalar denominator is nonzero.

(3) $${\hat{h}}_{t}=\frac{G_{r}b}{1+\Gamma H_{r,88}},\;\;H_{r,88}=b^{T}G_{r}b.$$

Here, ${\hat{h}}_{t}$ is the predicted target receptance column associated with a unit force at P8; its measured out-of-plane entries are displacement-to-force FRFs in m/N. The matrix $G_{r}$ is the reference receptance, b selects the P8 coordinate, and Γ is the gain in N/m. The scalar $H_{r,88}$ is the reference driving-point receptance in m/N. The two indices 8 identify the response and force coordinates at P8, respectively. The superscript T denotes transpose, the subscripts r and t identify the reference and target specimens, and the hat denotes a prediction. All quantities are evaluated at the same angular frequency. Equation (3) is the point-feedback specialization of established receptance modification methods [16,18].

Equating the predicted driving-point receptance to the measured target value gives the gain in Equation (4).

(4) $$\Gamma =\frac{1}{H_{t,88}}-\frac{1}{H_{r,88}}=i\omega \left(\frac{1}{Y_{t,88}}-\frac{1}{Y_{r,88}}\right).$$

Here, Γ is the equivalent displacement-feedback gain in N/m. The scalars $H_{t,88}$ and $H_{r,88}$ are the target and reference driving-point receptances in m/N, whereas $Y_{t,88}$ and $Y_{r,88}$ are the corresponding mobilities in (m/s)/N. The subscript pair 88 denotes response and excitation at P8; t and r denote the target and reference specimens. The angular frequency ω is expressed in rad/s, and i is the imaginary unit. Receptance and mobility satisfy Y=iωH under the adopted harmonic convention. Equation (4) applies where the driving-point FRFs are nonzero.

The gain represents the difference between the two condensed driving-point dynamic stiffnesses. It is not restricted to a real constant or interpreted as a physical spring stiffness. A velocity-feedback formulation would use the gain $\Gamma /\left(i\omega \right)$ in N s/m and yield the same FRF mapping when the conversion is applied consistently. Expressing the modified response in mobility form gives the spatial predictor in Equation (5).

(5) $${\hat{Y}}_{t,i8}=s_{r\to t}Y_{r,i8},\;\;s_{r\to t}=\frac{Y_{t,88}}{Y_{r,88}},\;\;i=1,\ldots ,15.$$

Here, ${\hat{Y}}_{t,i8}$ is the predicted target mobility at response point $P_{i}$ due to a force at P8, and $Y_{r,i8}$ is the measured reference mobility for the same transfer path. The dimensionless complex scalar $s_{r\to t}$ maps the reference response to the target response. It is calculated from the measured target and reference driving-point mobilities, $Y_{t,88}$ and $Y_{r,88}$. The subscripts t and r denote the target and reference specimens; the hat denotes a prediction. The response-point index i appears in the FRF subscripts; i in the mobility–receptance relation denotes the imaginary unit. All mobility terms have units of (m/s)/N and are evaluated at the same frequency, with $Y_{r,88}\neq 0$.

Only the target driving-point FRF is used to determine the gain. Responses at the remaining 14 target locations are withheld from calibration and used for spatial validation. Target coherence determines the eligibility of validation samples but does not enter the predictor. No modal parameter, mode label, or fitted spatial window is required. Equation (5) reproduces the target P8 response by construction. At every frequency, however, the predicted column remains proportional to the reference column. A complex gain can change the common amplitude and phase but cannot change the relative spatial pattern. Allowing the gain to vary with frequency does not remove this restriction. The formulation therefore provides spatial prediction conditioned on the measured target P8 FRF; it does not predict the response of an unmeasured target from fiber angle alone.

### 2.4. Relation to Anisotropic Reinforcement

Classical lamination theory gives the laminate bending stiffness coefficients through Equation (6) [4].

(6) $$D_{jk}\left(\theta \right)=\frac{1}{3}\sum_{l=1}^{N}{\bar{Q}}_{jk}^{\left(l\right)}\left(\theta \right)\left(z_{l}^{3}-z_{l-1}^{3}\right).$$

Here, $D_{jk}\left(\theta \right)$ is an entry of the laminate bending stiffness matrix at specimen fiber angle θ, with units of N m. The indices j,k∈{1,2,6} follow the in-plane component convention, in which 1, 2, and 6 correspond to the x, y, and xy components, respectively. The index l identifies a ply, and N is the total number of plies; N=12 for the CFRP specimens. The quantity ${\bar{Q}}_{jk}^{\left(l\right)}$ is an entry of the reduced stiffness matrix of ply l, transformed into the specimen axes, in Pa. The coordinates $z_{l-1}$ and $z_{l}$ locate its lower and upper interfaces relative to the laminate mid-plane and are expressed in meters. The summation includes all plies. The angle θ follows the clockwise convention from +y defined in Section 2.1; the corresponding lamina transformation angle measured counterclockwise from +x is $\alpha =90^{\circ }-\theta$.

For a uniform symmetric laminate, the thin-plate bending operator has the form given in Equation (7).

(7) $$\begin{array}{c} L_{D}w=D_{11}w_{,xxxx}+4D_{16}w_{,xxxy}+2\left(D_{12}+2D_{66}\right)w_{,xxyy}\\ +4D_{26}w_{,xyyy}+D_{22}w_{,yyyy}. \end{array}$$

Here, $L_{D}$ is the elastic bending operator constructed from the laminate bending stiffness matrix D, and $w=w\left(x,y\right)$ is the transverse displacement in meters. The coordinates x and y are the width and length directions, respectively, expressed in meters in this differential operator. A comma denotes partial differentiation: $w_{,xxxx}=\partial^{4}w/\partial x^{4}$, $w_{,xxxy}=\partial^{4}w/\left(\partial x^{3}\partial y\right)$, $w_{,xxyy}=\partial^{4}w/\left(\partial x^{2}\partial y^{2}\right)$, $w_{,xyyy}=\partial^{4}w/\left(\partial x\partial y^{3}\right)$, and $w_{,yyyy}=\partial^{4}w/\partial y^{4}$. The coefficients $D_{11}$ and $D_{22}$ describe bending stiffness in the two coordinate directions; $D_{12}$ couples their bending curvatures; $D_{66}$ describes twisting stiffness; and $D_{16}$ and $D_{26}$ describe bending–twisting coupling. All six coefficients have units of N m. The resulting quantity $L_{D}w$ is an elastic restoring load per unit area, in N/m^2^.

Off-axis reinforcement changes distributed bending stiffness, including bending–twisting coupling. In a discretized plate model, the stiffness change is assembled over the plate area from curvature interpolation matrices and changes in laminate bending coefficients. This is generally different from the single-coordinate update in Equation (2). Equation (5) is an established point-receptance rescaling identity, not a new representation of the distributed changes in D16 and D26. Mass, thickness, and dissipation can also affect the measured differences, particularly for the 3 mm matrix-only specimen. The available dimensions, masses, and nominal prepreg composition do not independently determine the lamina elastic and loss constants required by a validated numerical laminate model.

The inverse driving-point receptance is itself a condensed quantity. For an invertible block associated with the remaining coordinates, block elimination gives the Schur-complement expression in Equation (8).

(8) $$H_{88}^{-1}=Z_{88}-Z_{8q}Z_{qq}^{-1}Z_{q8}.$$

Here, $H_{88}$ is the driving-point receptance at P8, and $H_{88}^{-1}$ is its scalar reciprocal in N/m. The symbol q denotes the set of structural degrees of freedom other than the P8 out-of-plane coordinate. The scalar $Z_{88}$ is the P8 diagonal entry of the full dynamic stiffness matrix. The row block $Z_{8q}$ and column block $Z_{q8}$ couple P8 to the remaining coordinates, and $Z_{qq}$ is the dynamic stiffness submatrix for those coordinates. The superscript −1 on $Z_{qq}$ denotes a matrix inverse. All terms refer to the same specimen and frequency; the reference or target subscript is omitted because the identity applies to either specimen wherever the required inverses exist.

The frequency dependence of the condensed quantity includes the effects of the eliminated coordinates as well as inertia, stiffness, and dissipation. Frequency dependence of the gain is therefore consistent with dynamic condensation, but it does not establish frequency-dependent elastic reinforcement or uniquely separate stiffness and damping. Causality, passivity, and closed-loop stability constraints were not imposed on the gain calculated at each frequency. The formulation is consequently evaluated as a frequency-domain feasibility model, without claiming a physically realizable controller.

### 2.5. Data Eligibility, Validation Error, and Coverage

The primary magnitude-squared coherence threshold was $c_{min}=0.90$, and $c_{min}=0.95$ was used for sensitivity analysis. The symbol $c_{min}$ denotes a dimensionless data-quality threshold and is distinct from the geometric spacing c in Figure A2. At each frequency, the reference and target P8 coherences both had to meet the threshold. A validation output was retained only when its reference and target coherences also met the threshold. A frequency line was eligible when at least eight of the 14 validation outputs were retained. Responses with lower coherence were excluded without replacing them with zeros. Zero driving-point FRFs lie outside the domain of Equation (4).

Prediction accuracy was quantified using the pooled normalized complex FRF error defined in Equation (9).

(9) $$E_{B}={\left[\frac{\sum_{\omega \in \Omega_{B}}\sum_{i\neq 8}m_{i}\left(\omega \right){\left|{\hat{Y}}_{t,i8}\left(\omega \right)-Y_{t,i8}\left(\omega \right)\right|}^{2}}{\sum_{\omega \in \Omega_{B}}\sum_{i\neq 8}m_{i}\left(\omega \right){\left|Y_{t,i8}\left(\omega \right)\right|}^{2}}\right]}^{1/2}$$

Equation (9) sums squared complex residuals over the retained frequencies and validation outputs, excluding P8, and divides by the corresponding squared target-response norm. The result is dimensionless. The binary mask selects outputs meeting the mutual coherence criteria. The vertical bars denote complex modulus: for a complex number z, its modulus is the square root of the sum of the squares of its real and imaginary parts. A centered dot used inside modulus or norm notation elsewhere is an argument placeholder, not a time derivative. The outer square root converts the normalized squared residual to a relative norm error. All mobility terms in Equation (9) are evaluated at the same angular frequency and have units of (m/s)/N.

The set Ω_B_ contains the eligible angular-frequency samples in band B after the P8 coherence and minimum-output requirements are applied. The binary value m_i_(ω) is one when both specimens’ output-i coherences meet the threshold at that eligible sample, and zero otherwise. All arrays were finite and both P8 FRFs were nonzero; no extra direction-dependent exclusion was applied in the baseline. Reversing a reference–target pair leaves both frequency and output masks unchanged. Validation-cell coverage is the number of retained output–frequency cells divided by 14 times the band’s frequency-line count. It differs from frequency coverage and does not measure a fraction of continuous plate area.

Errors are reported as $100E_{B}$ percent. An error above 100% means that the residual norm exceeds the norm of the retained target response. Restricting Equation (9) to a single response location defines its pointwise error. Because pooling squared amplitudes assigns greater weight to stronger responses, pointwise errors are reported alongside the pooled metric. Frequency coverage was defined as the number of eligible frequency lines divided by the total number of lines in the evaluation band. It does not imply a continuous usable interval, and the retained outputs may vary between lines. The primary and secondary bands contained 2881 and 6400 lines, respectively. Ordinary reference–target comparisons used pairwise eligibility masks. Direct comparison of the 0° and 90° references for the 75° target additionally used an identical mask requiring valid data from all three specimens.

Measured coherence was used to assess data eligibility. Agreement between a prediction and a measured FRF was assessed separately using complex residuals and spatial correlation. For a fixed deterministic frequency-domain filter applied to one response channel, the filter factors cancel from magnitude-squared coherence wherever the filter is nonzero. Scalar transformation alone therefore does not demonstrate an improvement in measurement coherence.

### 2.6. Minimum Scalar Error and Spatial Correlation

Equation (10) defines the least-squares scalar and the RVAC for nonzero reference and target response vectors at an eligible frequency.

(10) $$s_{opt}=\frac{v_{r}^{H}v_{t}}{v_{r}^{H}v_{r}},\;\;\rho =\frac{{\left|v_{r}^{H}v_{t}\right|}^{2}}{\left(v_{r}^{H}v_{r}\right)\left(v_{t}^{H}v_{t}\right)}.$$

Here, $v_{r}$ and $v_{t}$ contain the mutually retained reference and target mobilities, respectively, at the validation locations, excluding P8. Their entries have units of (m/s)/N. The subscripts r and t denote the reference and target specimens. The dimensionless complex scalar $s_{opt}$ minimizes the Euclidean norm of the scaled-reference residual; the subscript opt denotes this optimum. The dimensionless real quantity $\rho \in \left[0,1\right]$ is the RVAC, expressed as a squared normalized complex correlation [20]. The superscript H denotes the Hermitian or conjugate transpose, and |⋅| denotes the complex modulus. Both vectors and the fitted scalar refer to the same frequency.

Projection onto the one-dimensional span of the reference vector gives the normalized minimum squared residual in Equation (11).

(11) $$\frac{∥s_{opt}v_{r}-v_{t}{∥}_{2}^{2}}{∥v_{t}{∥}_{2}^{2}}=1-\rho .$$

Here, $s_{opt}$ is the optimal complex scalar, and $v_{r}$ and $v_{t}$ are the nonzero reference and target mobility vectors over the retained validation locations. The subscripts r and t identify the specimens, and opt denotes the least-squares optimum. The notation $∥\cdot {∥}_{2}$ denotes the Euclidean norm, and ρ is the RVAC in Equation (10). Both sides are dimensionless. The corresponding minimum relative error at that frequency is $\sqrt{1-\rho }$. The bandwise minimum scalar error, denoted by $E_{opt,B}$, was obtained by using the optimal scalar at each frequency in Equation (9), with the same eligible outputs and lines as the P8-conditioned prediction. Its square is therefore the target-response-norm-weighted mean of 1−ρ, rather than a function of the median RVAC. By construction, $E_{opt,B}\leq E_{B}$.

The minimum scalar error requires the measured target responses at the validation locations. It is a retrospective least-squares compatibility measure, not an input available to a prospective one-point measurement procedure. It answers whether any scalar can match the measured response vectors on the retained samples. It does not separately identify measurement noise, support effects, or material mechanisms. A large minimum scalar error rules out a low-error scalar approximation on those samples; a small minimum with a larger P8-calibrated error indicates a mismatch between the driving-point ratio and the best spatial scalar.

For a fixed frequency and the same retained outputs, orthogonality of the least-squares residual gives the exact decomposition below. The bandwise terms use the same normalization and masks as Equation (9).

(12) $$∥v_{t}-s_{8}v_{r}{∥}_{2}^{2}=∥v_{t}-s_{opt}v_{r}{∥}_{2}^{2}+{\left|s_{8}-s_{opt}\right|}^{2}∥v_{r}{∥}_{2}^{2}.$$

(13) $$E_{B}^{2}=E_{opt,B}^{2}+E_{dp,B}^{2}.$$

Here, $s_{8}$ is the measured driving-point ratio, and $E_{dp,B}$ is the normalized contribution from its difference from the optimum spatial scalar. This term describes a mismatch between two scalar fits; it is not an estimate of instrument calibration error or statistical measurement uncertainty. For the 90–75° comparison in the primary band, the prediction error, minimum scalar error, and driving-point mismatch contribution are 18.16%, 16.55%, and 7.48%, respectively. Their squares satisfy Equation (13).

The squared driving-point mismatch term in Equation (13) is the sum, over the same eligible band samples, of the squared difference between the P8 ratio and the optimum spatial scalar multiplied by the retained reference-vector squared norm, divided by the retained target-vector squared norm summed over the band. Neither this term nor the minimum error statistically separates measurement variability from model discrepancy.

The Supplementary Materials present the reflection-parity diagnostic (Table S1 and Figure S1), complete CFRP reference–target results (Tables S2 and S3), valid-frequency support (Tables S4 and S5), processing sensitivity (Table S6), representative and complete upper-band results (Tables S7–S9), response weighting (Tables S10 and S11), additional output-count sensitivity (Tables S12a and S12b), and acquisition provenance (Table S13). Reflection parity is not used to estimate the gain or establish prediction accuracy.

### 2.7. Processing Sensitivity and Reference Comparison

All seven CFRP orientations were evaluated as references against the other six orientations, giving 42 directed comparisons per band. Self-comparisons were omitted. Each comparison used its own mutual coherence mask, with the same P8 calibration and 14 validation outputs. Matrix-only results were treated separately because of their different thickness. Reference rankings on different pairwise masks are descriptive; the 0° and 90° comparison for the 75° target additionally uses the identical three-specimen mask.

Processing sensitivity varied the minimum valid-output count over 4, 6, 8, 10, 12, and 14 at coherence thresholds 0.90 and 0.95. The principal comparison and the 0–15° and 0–30° cases were evaluated with the same rules. Eight outputs is an operational selection rule, not a physical threshold or optimized accuracy criterion. Minimum error was recalculated on every mask. For weak-driving-point exclusions, the P8 magnitude was compared with 1%, 2.5%, 5%, or 10% of the Euclidean norm of all 14 measured non-P8 FRFs at that frequency, before output coherence masking. Reference-only exclusions require no additional target responses; dual exclusions additionally use all 14 target outputs and are retrospective diagnostics. These checks change the selection of observations and do not provide confidence intervals or validate a prospective one-point acceptance rule.

For local validation, magnitude, real and imaginary components, wrapped phase difference, and absolute complex residual magnitude were plotted at representative low- and high-error locations. Phase difference was calculated as the argument of predicted mobility times conjugate measured mobility. Missing or excluded frequency samples were not interpolated. All calculations used the unsmoothed complex data and retained exact channel alignment.

For matched-mask benchmarks, the reference column was used unchanged, multiplied by one constant complex scalar per band, or multiplied by the frequency-dependent P8 ratio. The constant scalar minimizes the squared P8 residual over all band lines passing mutual P8 coherence, independent of the validation-output masks and minimum-output count. It uses the same reference and target P8 FRFs as the ratio predictor. All three predictions are evaluated on the identical baseline frequency/output mask. The per-frequency optimal spatial scalar additionally uses all retained target output amplitudes and is reported only as a retrospective lower bound. A zero-response prediction has exactly 100% error under Equation (9), providing a mathematical reference level rather than an engineering alternative.

## 3. Results

### 3.1. Availability of Response Data Meeting the Coherence Threshold

**Figure 2:**
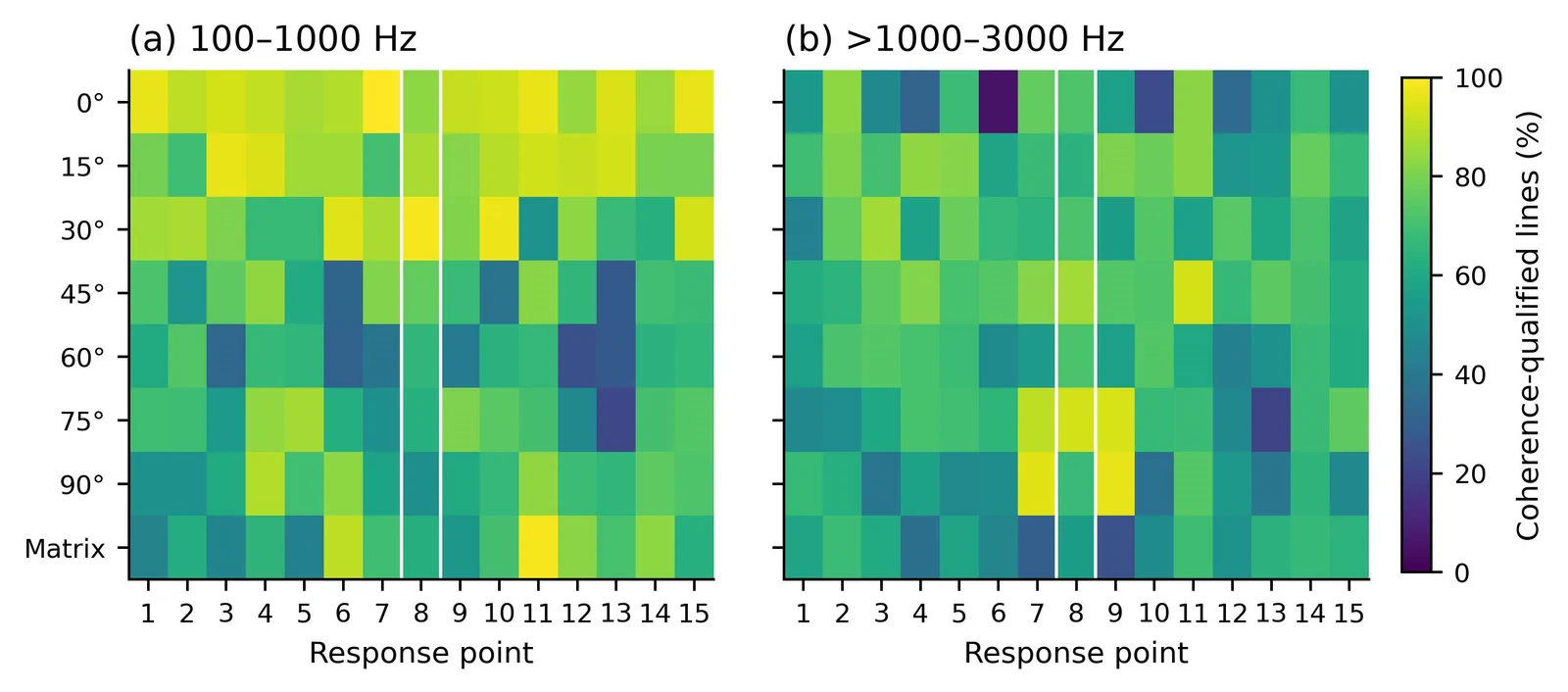
Frequency coverage at each response point for coherence ≥0.90: (**a**) 100 ≤ f ≤ 1000 Hz and (**b**) 1000 < f ≤ 3000 Hz. The white lines bracket P8. Coverage is calculated for individual channels before applying the mutual reference–target and minimum-output requirements.

Figure 2 shows the percentage of frequency lines meeting the 0.90 coherence threshold at each response point. Availability varied with specimen, location, and frequency band. In the primary band, at least 12 points met the threshold on 91.5% of lines for the 0° specimen and 20.7% for the 60° specimen. Requiring all 15 points reduced these percentages to 52.8% and 1.0%, respectively. The P8 channel met the threshold on 82.9% of lines for 0°, 98.6% for 30°, and 49.3% for 90°.

A prominent driving-point resonance does not ensure reliable responses at all validation locations. Requiring both the reference and target data to meet the eligibility criteria further reduces the frequency samples available for comparison. Pooled errors are therefore reported together with frequency coverage. Prediction accuracy is not inferred for excluded samples.

### 3.2. Predictions from the 0° Reference

**Figure 3:**
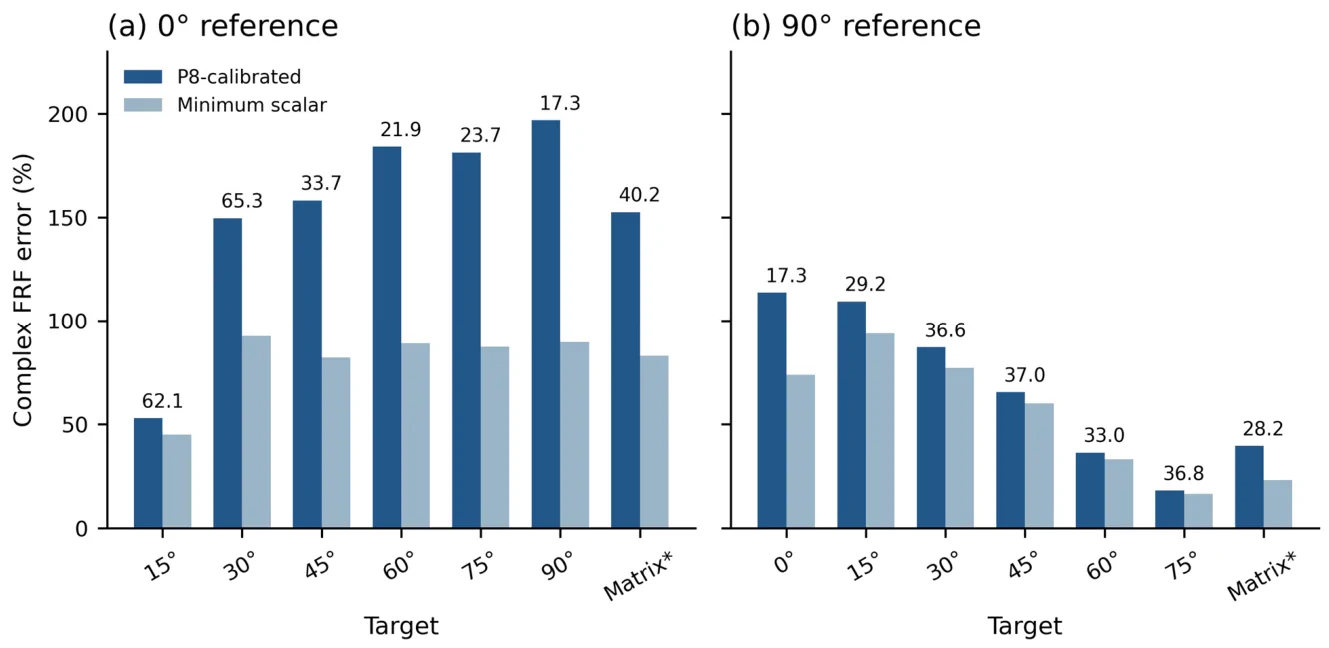
Pooled complex FRF prediction errors and minimum scalar errors within 100–1000 Hz using (**a**) the 0° reference and (**b**) the 90° reference. Predictions are conditioned on the target P8 FRF. Numbers above the bars denote frequency coverage (%). Each target uses its own pairwise mask at coherence ≥0.90; P8 is excluded from validation.

Table 2 and Figure 3a summarize the primary-band predictions obtained from the 0° reference. The 15° target had the lowest P8-conditioned error, 52.9%, and a minimum scalar error of 45.1%. For the other targets, prediction errors ranged from 149.7% to 196.9%, and minimum scalar errors ranged from 82.1% to 92.6%. The matrix-only specimen had a prediction error of 152.7% and a minimum scalar error of 83.0%. Thus, the discrepancies could not generally be explained by driving-point calibration alone. A substantial component of the retained target response remained outside the span of the reference response even when the optimal scalar was determined using the validation data.

Matrix rows are exploratory material-and-geometry comparisons: the matrix plate is 3.0 mm thick and does not match CFRP thickness.

The 30° target retained 65.3% of primary-band frequency lines but had a minimum scalar error of 92.6% and a median RVAC of 0.196. This discrepancy therefore persisted despite substantial frequency coverage. The 90° target combined a large error with limited coverage of 17.3%, so its result applies to a smaller subset of the band. Under these evaluation criteria, the 0° reference did not provide an accurate spatial approximation across the full range of orientations.

### 3.3. Dependence on Reference Orientation

The 0° and 90° specimens were initially used as physically interpretable axis-aligned references, not because intermediate references were unavailable. The revised assessment includes every CFRP orientation as a reference (Section 3.6). Table 2 and Table 3 retain the original endpoint comparisons. The 90°-reference results are also shown in Figure 3b. The 90° reference gave errors of 18.2% and 36.4% for the 75° and 60° targets, compared with 181.4% and 184.3% for the 0° reference on their respective pairwise masks. For 90–75°, the minimum scalar error was 16.5%. Different pairwise masks prevent these values from constituting a ranking on identical samples.

The matrix-only row is retained separately from the seven-orientation reference matrix because its thickness and mass differ.

To account for differences between pairwise masks, the two references were compared on identical samples meeting the eligibility criteria for the 0°, 90°, and 75° specimens. Within the primary band, this common set contained 418 lines, corresponding to 14.5% coverage. These lines were distributed between 100 and 473.125 Hz and did not constitute a continuous interval. On this set, the 0° reference gave a prediction error of 180.6% and a minimum scalar error of 94.5%; the corresponding values for the 90° reference were 17.3% and 15.8%. The advantage of the 90° reference therefore persisted on matched samples, although the directly comparable evidence was restricted to this low-frequency subset.

### 3.4. Spatial Variation in Prediction Accuracy

For 90–75°, the primary-band mask retained 1060 of 2881 lines (36.8%) in 20 disjoint intervals. The median RVAC was 0.973, and 79.8% of eligible lines had a spatial relative error of 30% or less. This descriptive percentage is not a statistical acceptance criterion. Pointwise errors ranged from 6.2% at P7 to 101.5% at P10, with a median of 22.5%; P6 had 91.3% error. Table A1 gives all pointwise errors and coverage. P6 and P10 together contributed only 0.43% of the retained squared target-response norm, so the amplitude-weighted pooled error understates their importance if these locations are critical. Giving the 14 pointwise errors equal weight produces a root-mean-square value of 45.9%; this is a separate location-weighting diagnostic on the existing pointwise masks, not the pooled error in Equation (9).

The retained set comprises 10,863 of 40,334 possible validation cells (26.93%), despite 36.79% frequency coverage. Eligible lines contain a mean of 10.25 retained outputs. Of the squared target-response amplitude used to normalize the pooled error, 96.83% lies in 200–300 Hz. The 18.2% value is therefore dominated by responses in that interval; it does not imply comparable accuracy across the rest of the primary band. Tables S10 and S11 give the exact point and frequency-bin weights. Excluded cells remain unevaluated rather than classified as accurate or inaccurate.

**Figure 4:**
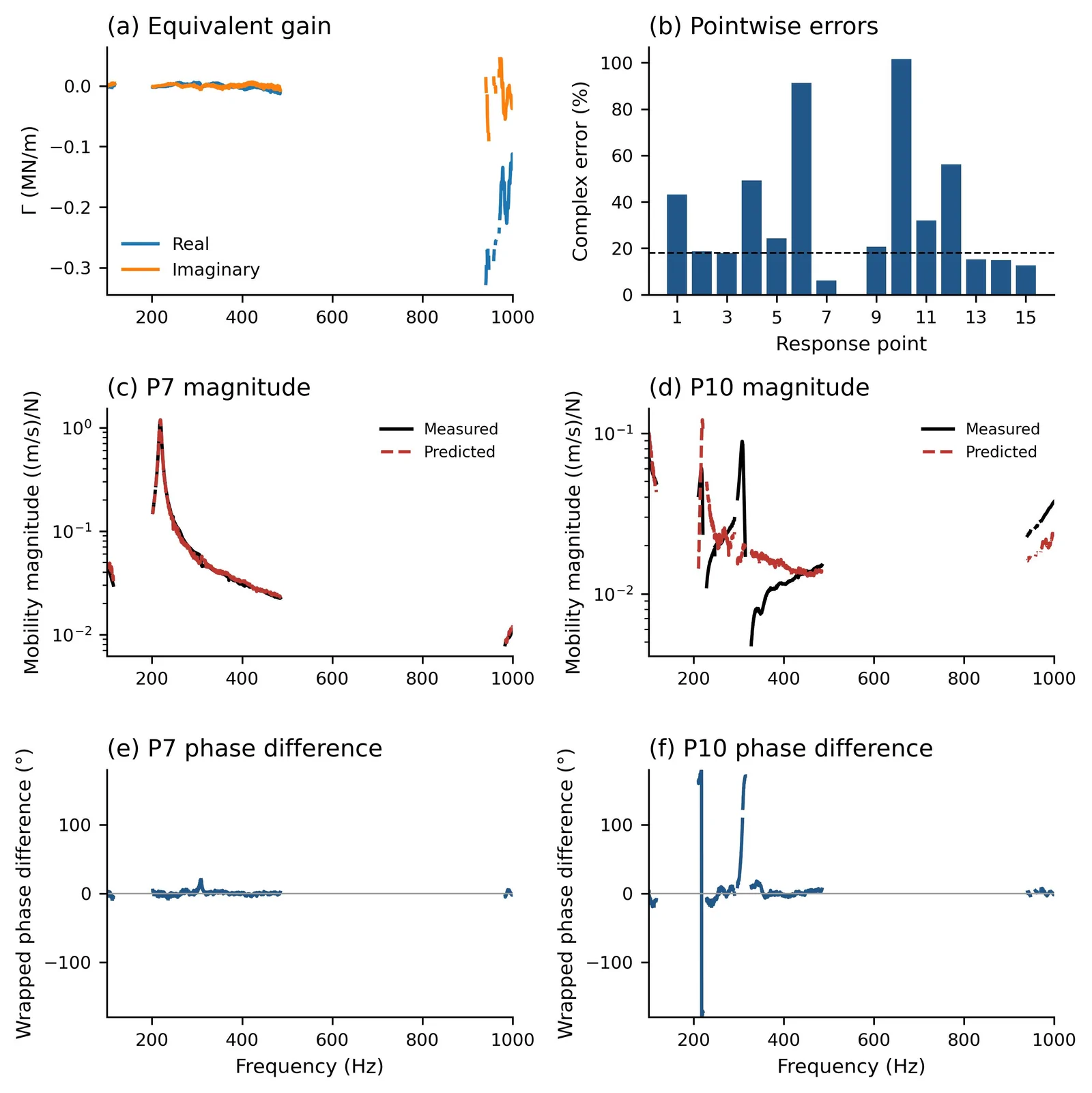
The 90–75° comparison within 100–1000 Hz: (**a**) real and imaginary parts of the equivalent gain; (**b**) pointwise complex errors with pooled error shown as a dashed line; (**c**,**d**) measured and predicted mobility magnitudes at P7 and P10; (**e**,**f**) wrapped prediction–measurement phase differences at the same locations. Only samples passing the relevant pair and point masks are plotted. Gaps are not interpolated. The dashed line is not a local acceptance tolerance.

Figure 4 summarizes the equivalent gain and local prediction results for 90°–75° within 100–1000 Hz. The equivalent gain describes a difference between condensed driving-point dynamic stiffnesses, not a directional modulus. Figure 4 shows both magnitude agreement and wrapped phase difference, calculated from the argument of the predicted response multiplied by the conjugate of the measured response. 

In the secondary band, 1000 < f ≤ 3000 Hz, the same pair gave a pooled prediction error of 98.4% and a minimum scalar error of 76.2%, at 36.2% coverage. The favorable primary-band result therefore did not extend to the higher band. The usefulness of a nearby reference orientation depends on spatial compatibility within the evaluation domain and the availability of reliable driving-point and validation measurements.

### 3.5. Sensitivity to Data Selection

Table 4 summarizes the sensitivity of the main comparisons to data selection. Increasing the coherence threshold from 0.90 to 0.95 reduced the 90–to-75° primary-band prediction error from 18.2% to 16.3%, while coverage decreased from 36.8% to 16.6%. The lower error indicates agreement on the more restricted set of retained samples, rather than improved accuracy on an unchanged validation set. The 90° reference also retained its advantage on the common three-specimen mask at 0.95, although only 98 lines, or 3.4% of the primary band, remained.

The absence of regularization in the baseline calculation was retained: no denominator clipping, smoothing, or gain fitting was applied. Prediction was calculated directly with the driving-point mobility ratio in Equation (5), rather than by numerically recombining the difference in reciprocals in Equation (4). Exactly zero driving-point FRFs lie outside the formulation and were not present in the supplied arrays. Sensitivity tests excluded relatively weak driving-point responses using thresholds of 1%, 2.5%, 5%, and 10% of the norm of the other 14 reference responses. Additional tests applied the same exclusion to both reference and target driving points (Supplementary Table S6). With a 5% reference-only threshold, the 0–15° error changed from 52.9% to 51.0%, while 0–30° remained 149.6%. The 90–75° result was unchanged at 18.2% and 36.8% coverage. These checks assess numerical sensitivity; they do not bound the uncertainty in the gain. Table 5 reports the effect of the minimum-output requirement for the 90°–75° comparison.

At coherence 0.90, requiring 4, 6, or 8 outputs gave errors near 18.1–18.2% and coverages of 41.0%, 38.0%, and 36.8%. Requiring 10, 12, or all 14 outputs increased errors to 20.3%, 31.9%, and 87.2%, while coverage fell to 21.5%, 7.4%, and 0.45%. At 0.95, all 14 outputs left no valid lines. Thus the favorable baseline does not establish broad plate-wide accuracy under stricter spatial completeness.

### 3.6. Complete CFRP Reference Comparison and Local Residuals

**Figure 5:**
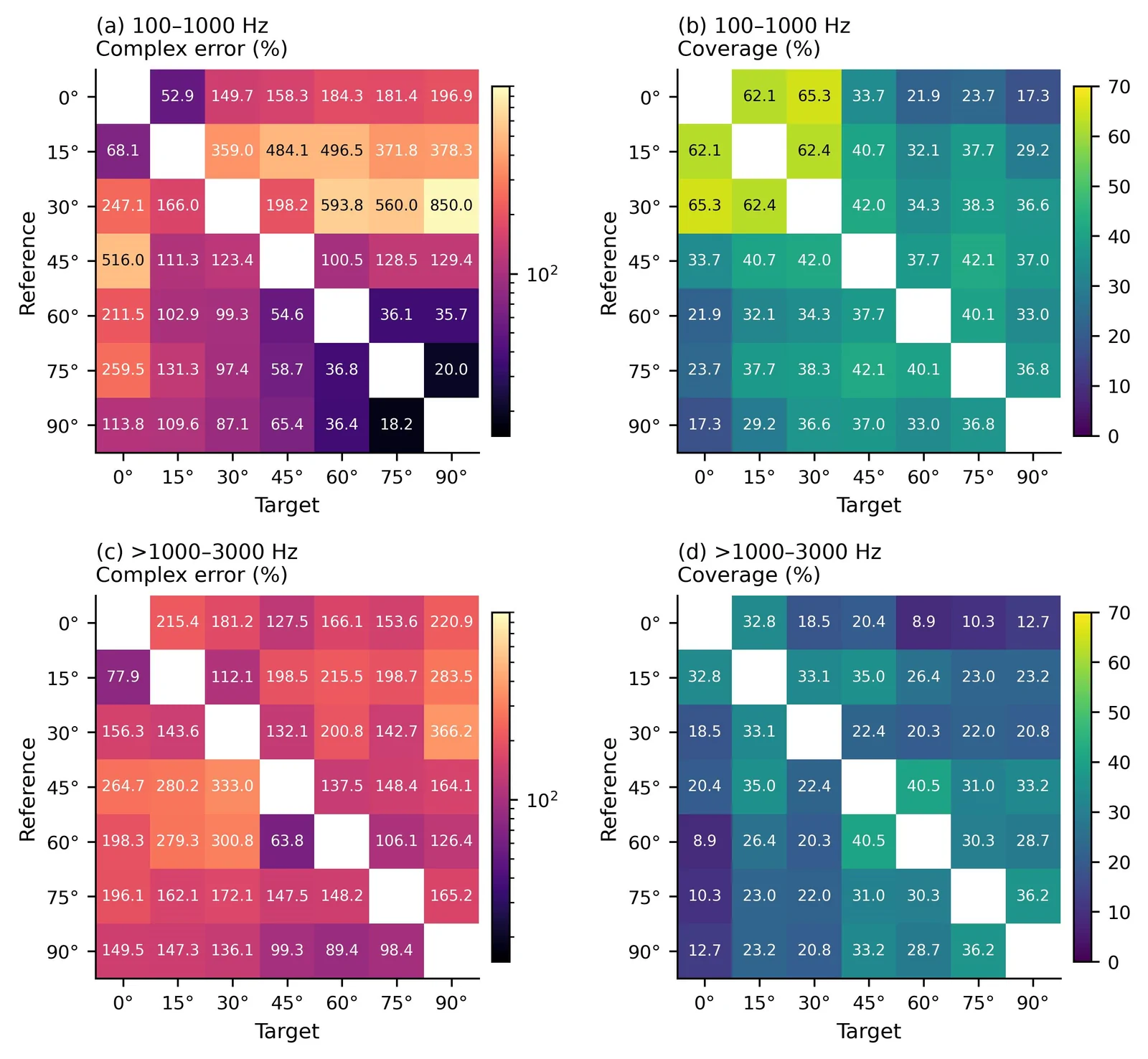
Complete CFRP reference–target matrices at coherence ≥0.90 with at least eight validation outputs: (**a**,**b**) primary-band complex error and coverage; (**c**,**d**) secondary-band complex error and coverage. Rows are references and columns are targets. Diagonal self-comparisons are omitted. Error colors use a logarithmic scale; cell numbers are percentages. Every cell uses its own pairwise mask, so this is not a ranking on common observations.

Figure 5 and Tables S2 and S3 report all 42 directed CFRP comparisons. In the primary band, 28 errors exceeded 100% and only two were at or below 30%; the latter descriptive threshold is not an application acceptance limit. In the secondary band, 37 exceeded 100% and none were at or below 30%. The primary-band minimum was 90–75°; the secondary-band minimum was 60–45° (63.8% at 40.5% coverage). These extrema are exploratory observations on pairwise masks using seven reused specimens, not independent demonstrations or a universal optimum reference. Most combinations do not support a low-error scalar approximation.

**Figure 6:**
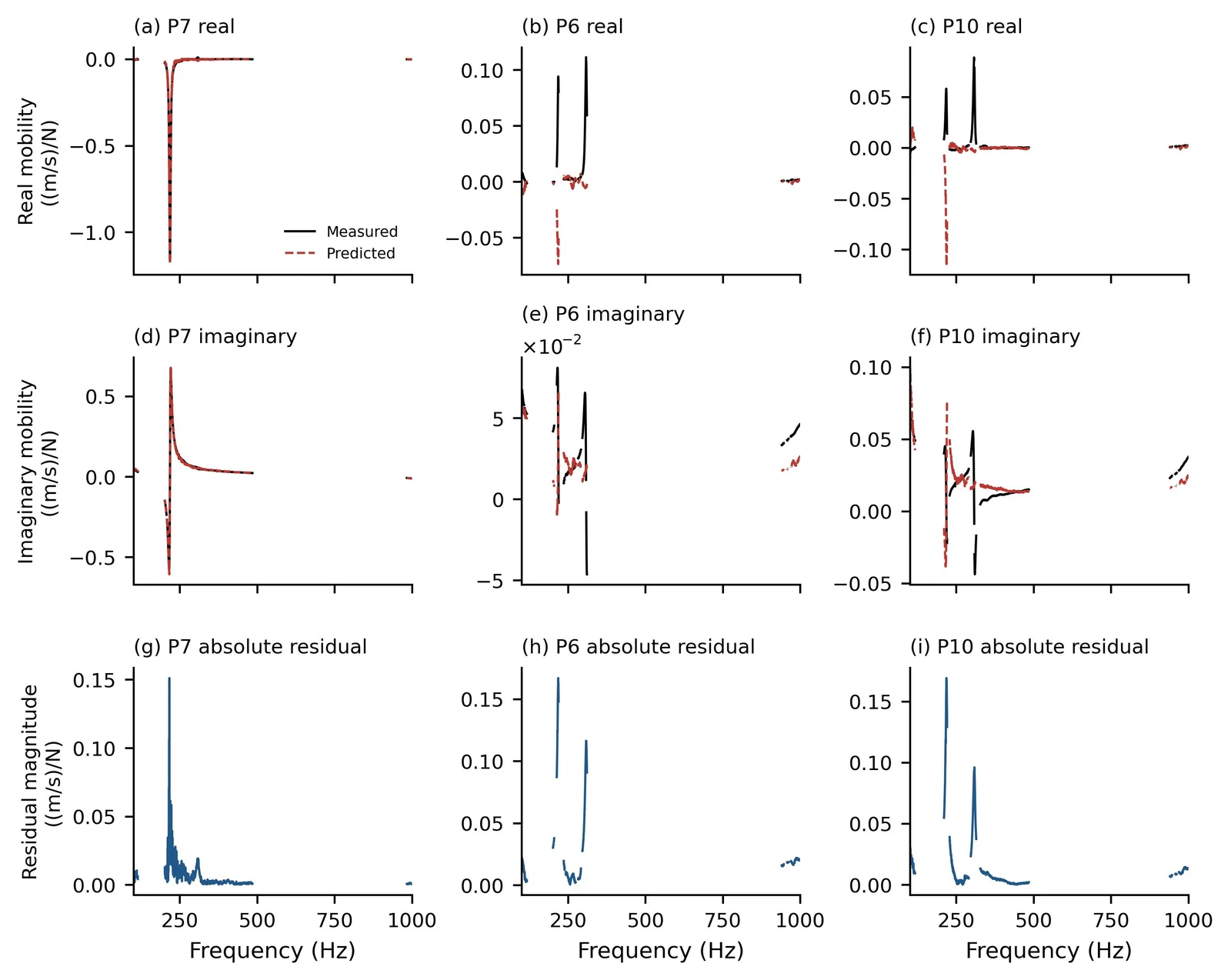
Local complex validation for the 90°–75° comparison within 100–1000 Hz: (**a**) real mobility at P7; (**b**) real mobility at P6; (**c**) real mobility at P10; (**d**) imaginary mobility at P7; (**e**) imaginary mobility at P6; (**f**) imaginary mobility at P10; (**g**) absolute complex residual magnitude at P7; (**h**) absolute complex residual magnitude at P6; (**i**) absolute complex residual magnitude at P10. Black solid and red dashed curves denote measured and predicted mobilities, respectively, and blue curves denote absolute complex residual magnitudes. Each location uses its pointwise mutual eligibility mask; excluded frequency samples are not interpolated.

Figure 6 compares P7, P6, and P10 on their respective valid samples. Real and imaginary residuals expose discrepancies that magnitude-only curves can conceal. Absolute residual magnitudes are reported in mobility units and complement relative errors, particularly at locations with weak target responses. A low pooled error is not a substitute for acceptable local response prediction.

### 3.7. Distribution of Valid Frequencies and Upper-Frequency Assessment

**Figure 7:**
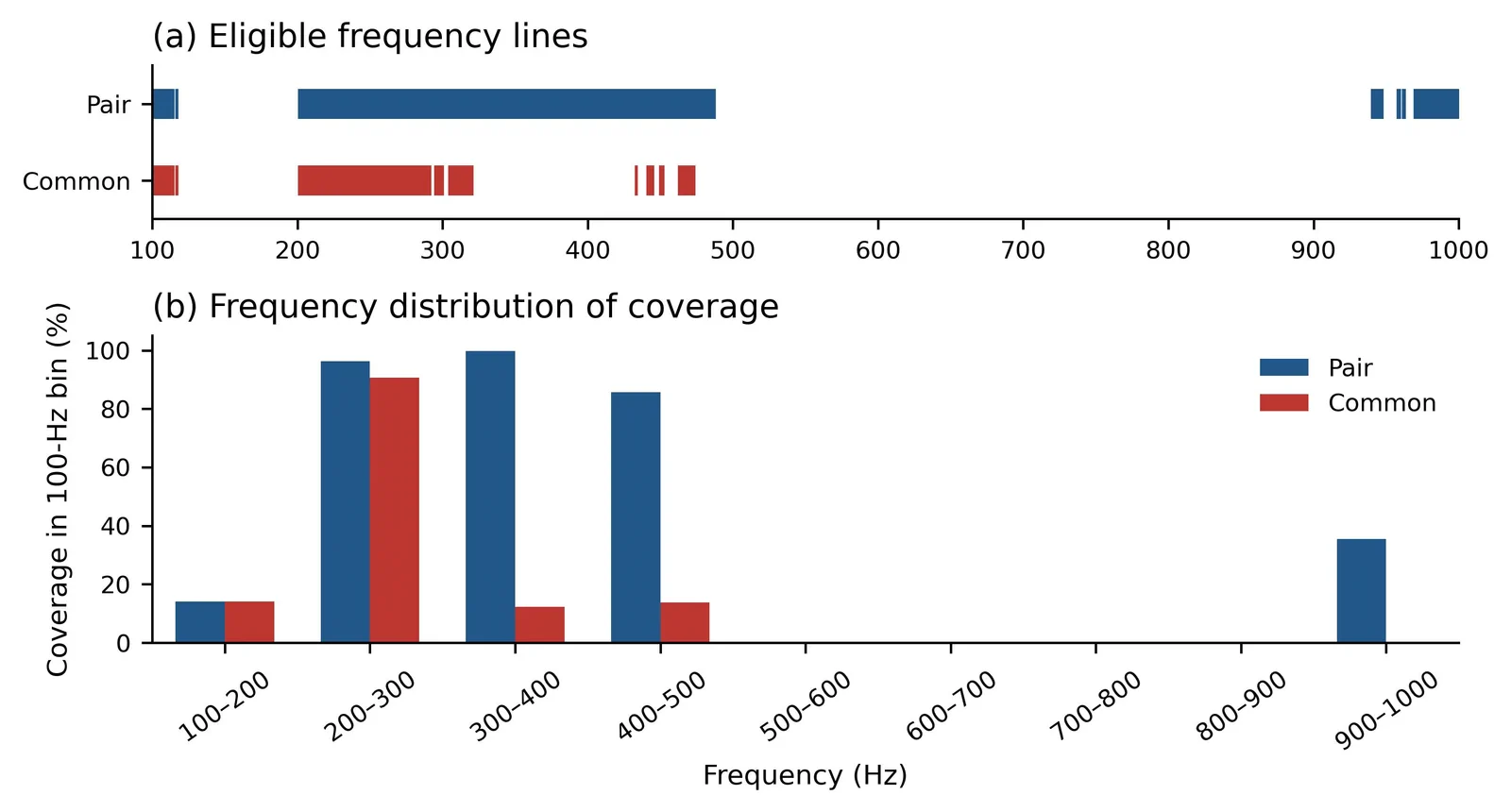
Frequency support of the 90–75° primary-band result: (**a**) individual eligible lines for the pair and common three-specimen masks; (**b**) coverage in 100-Hz bins. The last bin includes 1000 Hz. The two masks are not continuous bands.

Figure 7 and Supplementary Tables S4 and S5 identify the frequency support of the 18.2% result. The 20 retained intervals span 100–487.1875 Hz and 940.625–1000 Hz with gaps; no lines from 500 to below 900 Hz are retained. The two largest continuous intervals are 240.3125–308.4375 Hz (219 lines) and 309.0625–484.6875 Hz (563 lines). Of the 418 common-mask lines, 290 occur in 200–300 Hz. The common comparison is consequently concentrated at low frequency and cannot represent the entire primary band.

Extending the baseline rules gave 55.5% error and 13.6% coverage for 90–75° at 3000–5000 Hz, and 131.0% error and 4.0% coverage at 5000–8000 Hz. All 42 CFRP comparisons were eligible in 3000–5000 Hz; only 32 were eligible in 5000–8000 Hz, while 10 had no eligible lines. The five unordered pairs 45° with 0°, 15°, 30°, 60°, and 75° account for both directions of all 10 exclusions. Forward and reverse frequency/output masks were identical for every pair and band. Table S7 retains representative comparisons, and Tables S8 and S9 provide the complete tabulated upper-band results. Upper-band results remain descriptive because of the unresolved export bandwidth metadata in Section 2.2.

### 3.8. Comparison with Simpler Predictions

Table 6 compares representative cases on identical evaluation masks. For 90–75°, the frequency-dependent ratio reduced error from 45.3% without rescaling and 40.2% with a constant P8-fitted scalar to 18.2%. For 0–30°, however, its 149.7% error exceeded the constant-scalar error of 100.0%. Across the 42 primary-band comparisons, the ratio improved on unadjusted prediction in 26 cases and on the constant scalar in 14; the corresponding secondary-band counts were 19 and five. Frequency dependence therefore provides a measured benefit for some pairs but is not uniformly preferable. Table 6 reports the representative matched-mask benchmarks. Availability of additional supporting data is described in the Data Availability Statement and Supplementary Section S7.

The three prediction benchmarks use the reference column and at most the target P8 FRF. Eligibility of the reported evaluation cells still uses target output coherence; that domain is determined after full target measurement. Table 6 does not establish performance at outputs left unmeasured in a prospective application.

## 4. Discussion

### 4.1. Interpretation of the Frequency-Dependent Gain

Equation (4) determines the gain algebraically from two nonzero driving-point FRFs at each frequency, without mode tracking or fitting to the other response locations. Because an unconstrained complex gain has sufficient freedom to reproduce a single complex FRF exactly, agreement at P8 does not demonstrate that the reinforcement mechanism has been represented. The relevant evidence is the agreement at response locations excluded from gain estimation.

Classical lamination theory explains the spatial restriction of the scalar model. Changing fiber orientation alters distributed bending stiffness and coupling throughout the plate, whereas point feedback modifies one coordinate of the dynamic operator. These modifications may produce similar responses for a particular input and frequency range without implying equivalence of their structural operators. The present measurements show a restricted low-frequency approximation for one pair of nearby orientations, together with substantial discrepancies for other pairs and frequency bands.

The scalar gain can change the common amplitude and phase of the measured reference column, but cannot alter its relative spatial amplitudes and phases. The feedback interpretation therefore supplies a single-coordinate dynamic-stiffness representation; it does not encode the laminate bending matrix. The projection bound and decomposition in Equation (12) quantify measured response mismatch. These algebraic identities are established least-squares results, and their use here is diagnostic rather than a claim of a new theorem.

### 4.2. Practical Value and Relationship to Previous Modal Studies

The assessment identifies whether the measured response column admits a scalar approximation on a specified retained domain. The matched-mask benchmarks quantify whether the frequency-dependent P8 ratio adds practical benefit over leaving the reference response unchanged or fitting one constant scalar. The low error for 90–75° is accompanied by strong frequency weighting, limited validation-cell coverage, and large local discrepancies; most orientation pairs give much larger errors. If the minimum scalar error is large, changing only the scalar cannot provide a low-error fit on those observations. These are retrospective decisions using measured target responses and coherence, not online guarantees from P8 alone.

Previous tracking studies sought correspondence between identified modes or estimated modal system parameters [7,8,9]. The present analysis retains overlapping modal contributions in the complex forced response. It avoids explicit mode identification but cannot recover modes that the selected input fails to excite. A mode with zero normal displacement at the center has zero participation under an ideal central normal force; additional response locations alone cannot overcome this limitation.

A prospective measurement-saving procedure would require a reference and operating range validated on independent target measurements. The present study performs retrospective validation because full target FRFs were available. The P8 ratio itself uses only the target driving-point FRF, whereas RVAC, the optimum scalar, and the minimum error require target validation outputs. Consequently, these diagnostics must not be included in the information budget of a proposed one-point prediction. No independent prospective prediction trial was performed. Physics-informed reconstruction [11] and higher-rank receptance modification [19] use richer spatial information; their performance was not compared on this dataset.

### 4.3. Interpretation of the Matrix-Only Comparison

The matrix-only specimen used the same M.008 resin system and autoclave curing schedule, but had a measured thickness of 3.0 mm and mass of 44.3 g, compared with 1.9–2.1 mm and 37.3–37.5 g for the CFRP specimens. It is a separate material-and-geometry comparison, not a thickness-matched control for fiber reinforcement. Its FRF differences include geometry, mass, stiffness, and dissipation. Matrix results are retained as exploratory comparisons in Table 2 and Table 3, while the full orientation matrices in Figure 5 contain only CFRP specimens. No scalar thickness correction was applied because a thickness change can alter resonance locations and spatial responses. The shared curing schedule does not independently establish equal degree of cure or residual stress.

Modal damping ratios were not estimated, so the results do not test a specific relationship between damping and fiber angle. Establishing such a relationship would require modal or constitutive identification with quantified uncertainty. Complex FRFs retain the effects of dissipation but do not separate them from inertia and stiffness. This distinction is consistent with established composite damping formulations, in which directional material properties and modal deformation both influence dissipation [5,6].

### 4.4. Limitations and Requirements for Extension

The principal limitation is restricted observability. The measurements provide one input column of the transfer matrix and only the out-of-plane response at a sparse set of locations. They cannot uniquely determine the full mass, damping, or stiffness matrices, or identify a general matrix-valued feedback transformation between specimens. Reciprocity, where applicable, provides the corresponding row but not the remaining transfer paths. A model of distributed modification would require additional independent input locations or explicit structural assumptions supported by material and geometric characterization.

Each averaged FRF was obtained from ten impacts at one location on one tested specimen. Multiple coupons were manufactured from each parent panel, but independently tested specimen replicates and individual impact records were unavailable. Common-parent fabrication reduced separate-batch differences; local thickness and material variations and specimen-specific support contact remain possible confounders. The reported dimension and mass ranges do not quantify the dynamic effect of these differences. Statistical separation of measurement variability from model error is therefore not possible with the retained averages. Coherence, output-count, weighting, and driving-point sensitivity analyses evaluate processing dependence only [22]. Frequency lines and response points are not experimental replicates. The response-window decay of 100% adds no exponential damping, but finite-record leakage and input-window effects were not independently quantified.

The angle labels and specimen identities are confounded because one specimen represents each orientation. The full matrix cannot separate orientation effects from within-panel variation or specimen-specific support contact. The 42 directed comparisons reuse these seven specimens. The declared acquisition settings are based partly on the author’s account and were not independently audited against an original project; the export Span discrepancy is an additional provenance limitation for upper-band interpretation.

The valid-frequency masks restrict every comparison. For 90–75°, no primary-band lines from 500 to below 900 Hz were retained, and the common reference-comparison mask covers mainly 200–300 Hz. The 418-line common subset provides evidence for the reference comparison only on that subset; it is not representative of all 100–1000 Hz. Stricter output-count requirements substantially change error and coverage (Table 5). Local errors at P6 and P10 prevent a claim of uniformly accurate plate-wide prediction. Application-specific local tolerances and independent validation remain necessary.

The support arrangement was intended to approximate free–free conditions, but its stiffness and damping were not measured and altered-support tests were not performed. Foam contact can affect out-of-plane response even on an air-suspended rigid surface plate. The findings therefore apply to specimens tested in this common nominal support arrangement; they do not establish support-independent plate responses. Central excitation also limits observation of modes with zero or small normal displacement at P8. Neither high coherence nor additional output locations restores a mode that is not excited. Future work should assess support sensitivity, independent specimen and impact repeatability, and off-center excitation.

## 5. Conclusions

A scalar fictitious-feedback predictor was evaluated using 15-point SIMO mobility FRFs for seven CFRP orientations and a separate matrix-only specimen under nominally free–free conditions. The target driving-point FRF calibrated the gain, and the other response locations assessed prediction error. The matrix specimen shared the plan dimensions but differed in thickness and mass, so its comparison does not isolate fiber reinforcement.

The observed primary-band minimum was 18.2% for 90–75°, with 36.8% frequency coverage and 26.9% validation-cell coverage. Its pooled normalization was dominated by 200–300 Hz, which contributed 96.8% of retained squared target-response amplitude. Matched-mask errors of 45.3% without rescaling and 40.2% with a constant P8-fitted scalar demonstrate benefit for this case, while the ratio outperformed the constant scalar in only 14 of 42 primary-band comparisons. Local errors exceeded 90%, and stricter spatial completeness reduced coverage. These findings support a conditional retrospective assessment, not plate-wide or prospective accuracy.

The projection bound and driving-point mismatch partition the observed residual algebraically; they do not separate pure model error from measurement uncertainty. Most orientation pairs failed to yield low complex errors, and upper-band results further restricted the observed applicability. One tested specimen per orientation, unavailable individual impact records, unquantified support effects, unresolved acquisition metadata, and the matrix thickness mismatch limit physical attribution. Independent support tests, specimen replication, and prospective reference/domain validation are required before extending the conclusions beyond this measured configuration.

## Figures and Tables

**Table 1:** Measured specimen dimensions and masses. Observed ranges are reported for the CFRP specimens and for matrix plan dimensions; they are not standard deviations or confidence intervals.

Configuration	Width (mm)	Length (mm)	Thickness (mm)	Mass (g)
CFRP orientations	79.9–80.1	149.9–150.1	1.9–2.1	37.3–37.5
Matrix-only	79.9–80.1	149.9–150.1	3.0	44.3

**Table 2:** Spatial prediction errors using the 0° reference within 100–1000 Hz.

Target	Error (%)	Min. Scalar Error (%)	Coverage (%)	Median RVAC
15°	52.9	45.1	62.1	0.840
30°	149.7	92.6	65.3	0.196
45°	158.3	82.1	33.7	0.280
60°	184.3	89.0	21.9	0.285
75°	181.4	87.3	23.7	0.227
90°	196.9	89.7	17.3	0.108
Matrix	152.7	83.0	40.2	0.395

P8 is excluded from validation. Minimum scalar errors use the retained target validation responses and represent least-squares lower bounds. The coherence threshold is 0.90, with at least eight validation outputs required per eligible line.

**Table 3:** Spatial prediction errors using the 90° reference within 100–1000 Hz.

Target	Error (%)	Min. Scalar Error (%)	Coverage (%)
0°	113.8	73.7	17.3
15°	109.6	93.9	29.2
30°	87.1	77.1	36.6
45°	65.4	60.2	37.0
60°	36.4	33.1	33.0
75°	18.2	16.5	36.8
Matrix	39.7	23.3	28.2

Each row uses its own pairwise eligibility mask under the criteria in Table 2. Comparisons between rows therefore involve different retained samples.

**Table 4:** Sensitivity to data selection for the 75° target.

Ref.	Band (Hz)	$c_{min}$	Mask	$E_{B}$ (%)	Min. Error (%)	Cov. (%)
90°	100–1000	0.90	Pair	18.2	16.5	36.8
90°	100–1000	0.95	Pair	16.3	14.3	16.6
90°	>1000–3000	0.90	Pair	98.4	76.2	36.2
90°	>1000–3000	0.95	Pair	96.6	80.3	18.9
0°	100–1000	0.90	Common	180.6	94.5	14.5
90°	100–1000	0.90	Common	17.3	15.8	14.5
0°	100–1000	0.95	Common	191.2	92.3	3.4
90°	100–1000	0.95	Common	15.0	12.1	3.4

Pair denotes the reference–target mask; Common denotes the identical 0°/90°/75° mask. $c_{min}$ is the coherence threshold, $E_{B}$ is the pooled complex error, Min. error is the minimum scalar error, and Cov. is frequency coverage. Changing the threshold also changes the retained samples.

**Table 5:** Minimum-output-count sensitivity for 90–75° within 100–1000 Hz. P8 is excluded from the output count. Each row has a different mask; errors therefore do not describe the same observations. An em dash indicates no eligible lines.

Coherence	Min. Outputs	Eligible Lines	Error (%)	Min. Error (%)	Coverage (%)
0.90	4	1180	18.1	16.5	41.0
0.90	6	1094	18.1	16.5	38.0
0.90	8	1060	18.2	16.5	36.8
0.90	10	619	20.3	18.4	21.5
0.90	12	214	31.9	30.2	7.4
0.90	14	13	87.2	86.0	0.5
0.95	4	793	16.8	14.9	27.5
0.95	6	760	16.7	14.9	26.4
0.95	8	478	16.3	14.3	16.6
0.95	10	90	15.9	13.0	3.1
0.95	12	4	18.1	14.8	0.1
0.95	14	0	—	—	0.0

**Table 6:** Complex errors (%) for matched-mask benchmarks. The constant scalar is fitted only to mutually coherent P8 data; the spatial optimum uses withheld target outputs retrospectively.

Pair	Band (Hz)	Unadjusted	Constant P8 fit	P8 Ratio	Spatial Optimum
90° → 75°	100–1000	45.3	40.2	18.2	16.5
0° → 15°	100–1000	144.0	99.6	52.9	45.1
0° → 30°	100–1000	205.4	100.0	149.7	92.6
60° → 45°	>1000–3000	127.5	94.1	63.8	54.5

## Data Availability

The data presented in this study are included in the article and its Supplementary Materials. Additional supporting data, where available, may be obtained from the corresponding author upon reasonable request.

## Supplementary Materials

The following supporting information can be downloaded at: https://www.mdpi.com/article/10.3390/polym18212631/s1, Figure S1. Reflection-parity squared-response fractions for (a) 100–1000 Hz and (b) 1000 < f ≤ 3000 Hz. Numbers above bars give frequency coverage (%) at coherence ≥0.90. Specimen-specific masks retain complete reflection orbits and at least eight points. Table S1. Reflection-parity fractions within 100–1000 Hz. Table S2. CFRP reference–target results within 100–1000 Hz. Table S3. CFRP reference–target results within 1000 < f ≤ 3000 Hz. Table S4. All eligible intervals for 90°–75° within 100–1000 Hz. Endpoints are included; spacing is 0.3125 Hz. Table S5. Pairwise and common 0°/90°/75° frequency support. Upper bin endpoints are excluded except at 1000 Hz. Table S6. Weak-driving-point sensitivity within 100–1000 Hz. Coherence ≥0.90 and at least eight outputs are required. At zero threshold, the reference-only baseline also applies to the dual test. Table S7. Representative comparisons across frequency bands. Masks differ by band. A dash indicates undefined error when no lines are eligible. Table S8. All 42 directed CFRP comparisons within 3000 < f ≤ 5000 Hz. Table S9. All 42 directed CFRP comparisons within 5000 < f ≤ 8000 Hz. Table S10. Exact point weights for 90°–75° within 100–1000 Hz. Squared amplitudes are not mechanical energy. Table S11. Frequency-bin weighting for the same comparison. Upper endpoints are excluded except at 1000 Hz. Table S12a. Selected output-count and coherence sensitivity results for 0°–15° within 100–1000 Hz. Table S12b. Selected output-count and coherence sensitivity results for 0°–30° within 100–1000 Hz. Table S13. Essential acquisition information and traceability limits.

## Appendix A. Measurement Coordinates and Pointwise Validation

Table A1 lists the nominal measurement coordinates and pointwise errors for the 90°-to-75° comparison. Coordinates are expressed in millimeters. Pointwise coverage is calculated relative to all 2881 lines in the primary band. Each location is evaluated only where the pairwise frequency-line criteria and its own mutual coherence requirement are satisfied. P8 is used for calibration and is excluded from the validation statistics.

**Table A1:** Coordinates and pointwise 90°-to-75° validation within 100–1000 Hz.

Point	x (mm)	y (mm)	Error (%)	Coverage (%)
P1	0	0	43.2	13.9
P2	40	0	18.7	12.4
P3	80	0	17.9	17.3
P4	80	37.5	49.3	36.1
P5	40	37.5	24.3	33.8
P6	0	37.5	91.3	12.9
P7	0	75	6.2	34.5
P8	40	75	Calibration	—
P9	80	75	20.7	33.2
P10	80	112.5	101.5	32.5
P11	40	112.5	32.1	36.2
P12	0	112.5	56.2	28.6
P13	0	150	15.3	12.3
P14	40	150	14.9	36.7
P15	80	150	12.7	36.8

## Appendix B. Specimen Photographs and Measurement Schematic

Figure A1 shows representative CFRP and matrix-only specimens on foam, and Figure A2 gives plan dimensions and thickness distinctions. Both appendices are referenced in Section 2.1 and Section 2.2. The photographs document the nominal support arrangement; they do not establish negligible support stiffness or damping.

## References

[1] Guo R., Li C., Niu Y., Xian G. (2022). The fatigue performances of carbon fiber reinforced polymer composites—A review. J. Mater. Res. Technol..

[2] Xian G., Guo R., Li C., Wang Y. (2022). Mechanical performance evolution and life prediction of prestressed CFRP plate exposed to hygrothermal and freeze-thaw environments. Compos. Struct..

[3] Yu Y., Pan Y., Zhou R., Miao X. (2021). Effects of water and alkaline solution on durability of carbon-glass hybrid fiber reinforced polymer bars. Polymers.

[4] Nettles A.T. (1994). Basic Mechanics of Laminated Composite Plates.

[5] Lin D.X., Ni R.G., Adams R.D. (1984). Prediction and measurement of the vibrational damping parameters of carbon and glass fibre-reinforced plastics plates. J. Compos. Mater..

[6] Sol H., Rahier H., Gu J. (2020). Prediction and measurement of the damping ratios of laminated polymer composite plates. Materials.

[7] Kim C.-J. (2021). Modal parameter tracking in a carbon fiber-reinforced structure over different carbon fiber angles. J. Mar. Sci. Eng..

[8] Kim C.-J. (2021). Prediction of system parameters of carbon-based composite structure for different carbon fiber orientations with mode information at reference angle only. Materials.

[9] Kim C.-J. (2022). Mode tracking of unidirectional carbon-based composite structures using modified mode shape vectors. Crystals.

[10] Milewski M. (2026). Influence of local fiber orientation deviations on the dynamic and mechanical response of CFRP laminates for UAV structures. Fibers.

[11] Hedayatrasa S., Fink O., Van Paepegem W., Kersemans M. (2025). k-space physics-informed neural network (k-PINN) for compressed spectral mapping and efficient inversion of vibrations in thin composite laminates. Mech. Syst. Signal Process..

[12] Zanetti M., Negri D., Vandepitte D., Tita V., De Medeiros R. (2025). Analysing the influence of delamination on composite structures using modal analysis and statistical indices. Compos. Struct..

[13] Peeters B., Van der Auweraer H., Guillaume P., Leuridan J. (2004). The PolyMAX frequency-domain method: A new standard for modal parameter estimation?. Shock Vib..

[14] Rothberg S.J., Allen M.S., Castellini P., Di Maio D., Dirckx J.J.J., Ewins D.J., Halkon B.J., Muyshondt P., Paone N., Ryan T. (2017). An international review of laser Doppler vibrometry: Making light work of vibration measurement. Opt. Lasers Eng..

[15] Wróbel J., Jendryka K., Milewski M., Kierzkowski A., Stosiak M., Prentkovskis O., Karpenko M. (2026). Experimental modal testing of lightweight composite UAV structures: Methods and key challenges. Machines.

[16] Ram Y.M., Mottershead J.E. (2007). Receptance method in active vibration control. AIAA J..

[17] Ahn H.-J., Kim C.-J. (2016). Frequency domain indirect identification of AMB rotor systems based on fictitious proportional feedback gain. J. Mech. Sci. Technol..

[18] Ouyang H., Wei X., Mottershead J.E. (2025). Structural modification and active vibration control by the receptance method: Review and tutorial. Mech. Syst. Signal Process..

[19] Shi Y., Li S. (2026). Receptance-based higher-rank structural modification for multi-frequency radiated sound power control. Comput. Struct..

[20] European Cooperation for Space Standardization Response Vector Assurance Criterion RVAC; ECSS-E-ST-32-11C Glossary Entry. https://ecss.nl/item/?glossary_id=327.

[21] Siemens Digital Industries Software Simcenter Testlab Impact Testing. https://community.sw.siemens.com/articles/en_US/Knowledge/simcenter-testlab-impact-testing.

[22] Bendat J.S., Piersol A.G. (2010). Random Data: Analysis and Measurement Procedures.

