# Supplementary material for: Spatial Applicability of Fictitious Point Feedback for Frequency Response Prediction in Unidirectional Carbon Fiber-Reinforced Polymer Plates
Source: Polymers (Basel). 2026 Oct 23;18(21):2631. doi: 10.3390/polym18212631 (PMC13766004; doi:10.3390/polym18212631)
Supplement: Supplementary file 1 [file polymers-18-02631-s001.zip › polymers-4622049-supplementary.pdf]

Supplementary Materials

Spatial Applicability of Fictitious Point Feedback for Frequency Response Prediction in Unidirectional Carbon Fiber-Reinforced Polymer Plates

This file provides the optional reflection-parity diagnostic and the numerical sensitivity and reference-comparison results supporting the revised manuscript. Parity does not determine the feedback gain or establish predictive accuracy. All added material is shown in red relative to the original supplement.

S1. Reflection-parity diagnostic

The symmetric measurement grid permits a decomposition by reflection parity without identifying individual modes. The four orthogonal projection matrices are defined in Equation (S1).

$$\mathbf{P}_{\sigma\tau} = \frac{1}{4}(\mathbf{I} + \sigma\mathbf{R}_x)(\mathbf{I} + \tau\mathbf{R}_y), \qquad \sigma, \tau \in \{+1, -1\}. \tag{S1}$$

Here, $\mathbf{P}_{\sigma\tau}$ is a dimensionless 15 × 15 matrix that projects a sampled response onto one reflection-parity subspace, and $\mathbf{I}$ is the identity matrix of the same size. The dimensionless permutation matrices $\mathbf{R}_x$ and $\mathbf{R}_y$ exchange measurement points under reflection in the width and length directions, respectively. With coordinates in millimeters, these mappings are $(x, y) \mapsto (80 - x, y)$ and $(x, y) \mapsto (x, 150 - y)$. The coordinates x and y denote the width and length positions. The signs σ and τ specify parity under the respective reflections: +1 denotes an even component and −1 an odd component. The reflection matrices commute, and applying either reflection twice gives the identity.

The components are even–even (++), even–odd (+−), odd–even (−+), and odd–odd (−−). Each retained point and all its reflected counterparts had to meet the coherence threshold, with at least eight of the 15 points retained. Complete reflection orbits preserve the sampling-mask symmetries and projection orthogonality. These specimen-specific masks were constructed independently of the prediction masks.

For each parity component, Equation (S2) defines its aggregate squared-response fraction over the evaluation band.

$$\eta_{\sigma\tau,B} = \frac{\sum_{\omega\in\Omega_B^{\mathrm{par}}} \|\mathbf{W}(\omega)\mathbf{P}_{\sigma\tau}\mathbf{y}(\omega)\|_2^2}{\sum_{\omega\in\Omega_B^{\mathrm{par}}} \|\mathbf{W}(\omega)\mathbf{y}(\omega)\|_2^2}. \tag{S2}$$

Here, $\eta_{\sigma\tau,B}$ is the dimensionless fraction associated with parity signs σ and τ in frequency band B. Each sign is +1 for even parity or −1 for odd parity under the width and length reflections, respectively. The set Ω_B^{par} contains the eligible angular-frequency samples for the parity analysis of the specimen; the superscript par distinguishes this set from the pairwise prediction set in Equation (9) of the main manuscript. The symbol ω denotes angular frequency. The vector $\mathbf{y}(\omega)$ contains all 15 measured out-of-plane mobilities for central excitation, in (m/s)/N. It is distinct from the reduced validation vectors in Equation (10) of the main manuscript. The diagonal 15 × 15 matrix $\mathbf{W}(\omega)$ has entries of one for points belonging to retained complete reflection orbits and zero otherwise. The matrix $\mathbf{P}_{\sigma\tau}$ is the parity projector in Equation (S1), and $\|\cdot\|_2$ denotes the Euclidean norm. The sums aggregate the squared norms over the eligible frequency samples.

The fractions sum to one and quantify squared response amplitudes, not energy, modal participation, or material anisotropy. Ideal central normal excitation of a rectangular isotropic or axis-aligned orthotropic plate with symmetric free boundaries produces an even–even response. Off-axis coupling in Equation (7) of the main manuscript permits an odd–odd component while preserving half-turn symmetry. Mixed parity departs from that ideal symmetry. Supports, force placement, material nonuniformity, and measurement error can also change these fractions; the diagnostic does not identify their cause. The matrix-only specimen is thicker than the CFRP specimens; its parity fractions remain descriptive sampled-response fractions, not a thickness-matched material comparison.

S2. Descriptive results and limitations

Table S1 and Figure S1 summarize specimen-specific response fractions. Axis-aligned and matrix-only specimens were predominantly even–even in the primary band, whereas several off-axis specimens had appreciable odd–odd components. Frequencies retained for each specimen differ.

Table S1. Reflection-parity fractions within 100–1000 Hz.

Specimen	Even–even (%)	Odd–odd (%)	Mixed (%)	Coverage (%)
0°	99.68	0.10	0.21	91.6
15°	85.66	13.87	0.46	78.1
30°	44.33	50.25	5.41	61.6
45°	72.63	25.21	2.15	27.5
60°	90.17	8.14	1.69	21.4
75°	98.20	1.12	0.68	24.5
90°	98.99	0.16	0.85	58.3
Matrix	98.49	0.22	1.30	39.5

Fractions describe squared sampled-response norms and do not represent mechanical energy. Mixed parity combines +− and −+. Each specimen uses a mask of complete reflection orbits with coherence ≥ 0.90 and at least eight retained points.

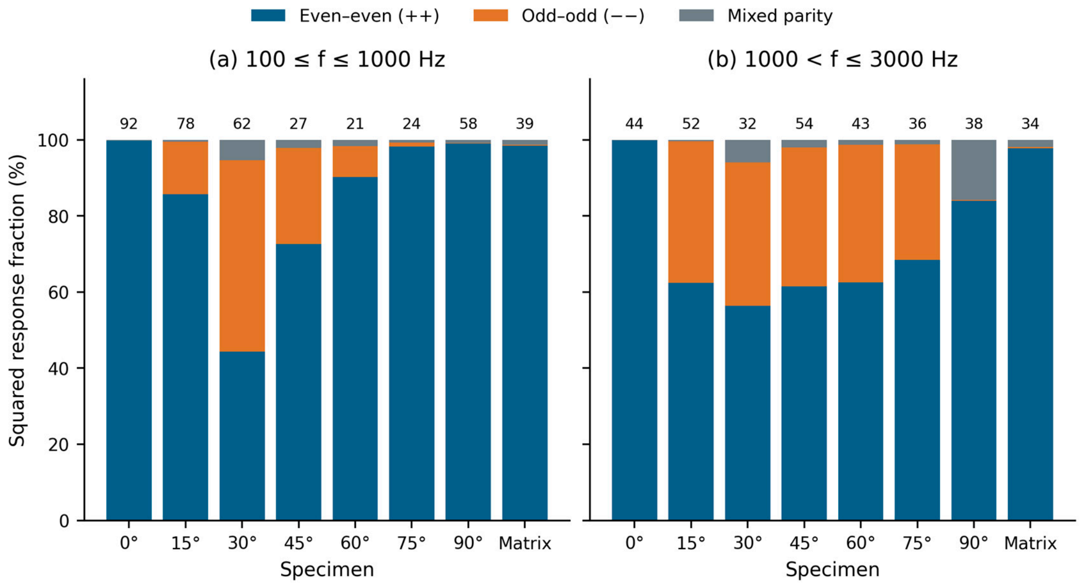

Figure S1. Squared-response fractions by reflection parity: (a) 100 ≤ f ≤ 1000 Hz and (b) 1000 < f ≤ 3000 Hz. Mixed parity combines +− and −+. Numbers above the bars indicate frequency coverage (%) for specimen-specific masks of complete reflection orbits at coherence ≥ 0.90. The retained frequencies differ between specimens, so the fractions do not represent material properties evaluated on common samples.

Scalar multiplication preserves relative parity content, but equal parity fractions do not imply spatial collinearity, as illustrated by the poor 0°-to-90° prediction. Parity is therefore a supplementary diagnostic, not a criterion for accepting a prediction.

The 90° specimen had 15.8% mixed parity in the secondary band, decreasing to 14.6% at coherence ≥ 0.95. This departure from ideal half-turn symmetry cannot be uniquely attributed to material anisotropy: support asymmetry, force-placement error, specimen variability, and measurement effects remain possible causes.

A common complete-orbit mask across all eight specimens retained only 33 primary-band lines (1.15%) and two secondary-band lines (0.031%) at coherence ≥ 0.90, and none at 0.95. The specimen-specific fractions

consequently do not establish an orientation-dependent material law on common frequency samples. They are not used as an anisotropy index, a gain-selection rule, or independent evidence of predictive validity.

S3. Complete CFRP reference–target results

The following matrices use coherence ≥ 0.90 and at least eight of the 14 validation outputs, with mutual P8 coherence required. Rows denote reference orientation and columns target orientation. Each cell has its own frequency/output mask; diagonal self-comparisons are omitted. The matrix-only plate is excluded from orientation matrices because its 3.0 mm thickness differs from the CFRP thickness. The original exploratory matrix comparisons remain in main manuscript Tables 2 and 3.

Table S2. CFRP reference–target results within 100–1000 Hz.

P8-calibrated complex error (%)

Ref. / target	0°	15°	30°	45°	60°	75°	90°
0°	—	52.9	149.7	158.3	184.3	181.4	196.9
15°	68.1	—	359.0	484.1	496.5	371.8	378.3
30°	247.1	166.0	—	198.2	593.8	560.0	850.0
45°	516.0	111.3	123.4	—	100.5	128.5	129.4
60°	211.5	102.9	99.3	54.6	—	36.1	35.7
75°	259.5	131.3	97.4	58.7	36.8	—	20.0
90°	113.8	109.6	87.1	65.4	36.4	18.2	—

Minimum scalar error (%)

Ref. / target	0°	15°	30°	45°	60°	75°	90°
0°	—	45.1	92.6	82.1	89.0	87.3	89.7
15°	55.6	—	90.2	51.8	69.9	77.7	91.1
30°	91.6	86.4	—	94.6	83.3	69.5	64.9
45°	82.8	53.1	98.0	—	38.9	44.6	59.2
60°	59.8	82.3	90.3	40.6	—	32.9	34.0
75°	57.7	92.6	78.6	48.2	33.2	—	15.5
90°	73.7	93.9	77.1	60.2	33.1	16.5	—

Frequency coverage (%)

Ref. / target	0°	15°	30°	45°	60°	75°	90°
0°	—	62.1	65.3	33.7	21.9	23.7	17.3
15°	62.1	—	62.4	40.7	32.1	37.7	29.2
30°	65.3	62.4	—	42.0	34.3	38.3	36.6
45°	33.7	40.7	42.0	—	37.7	42.1	37.0
60°	21.9	32.1	34.3	37.7	—	40.1	33.0
75°	23.7	37.7	38.3	42.1	40.1	—	36.8

Ref. / target	0°	15°	30°	45°	60°	75°	90°
90°	17.3	29.2	36.6	37.0	33.0	36.8	—

Table S3. CFRP reference–target results within 1000 < f ≤ 3000 Hz.

P8-calibrated complex error (%)

Ref. / target	0°	15°	30°	45°	60°	75°	90°
0°	—	215.4	181.2	127.5	166.1	153.6	220.9
15°	77.9	—	112.1	198.5	215.5	198.7	283.5
30°	156.3	143.6	—	132.1	200.8	142.7	366.2
45°	264.7	280.2	333.0	—	137.5	148.4	164.1
60°	198.3	279.3	300.8	63.8	—	106.1	126.4
75°	196.1	162.1	172.1	147.5	148.2	—	165.2
90°	149.5	147.3	136.1	99.3	89.4	98.4	—

Minimum scalar error (%)

Ref. / target	0°	15°	30°	45°	60°	75°	90°
0°	—	57.2	70.4	76.4	77.5	91.6	80.6
15°	76.7	—	58.1	79.3	74.5	94.8	91.7
30°	98.0	87.5	—	99.5	97.4	94.3	91.7
45°	68.6	90.2	97.9	—	54.6	83.6	93.1
60°	76.0	76.1	92.7	54.5	—	73.3	90.0
75°	91.1	98.8	96.7	92.2	67.6	—	85.2
90°	80.9	97.2	98.1	95.0	85.8	76.2	—

Frequency coverage (%)

Ref. / target	0°	15°	30°	45°	60°	75°	90°
0°	—	32.8	18.5	20.4	8.9	10.3	12.7
15°	32.8	—	33.1	35.0	26.4	23.0	23.2
30°	18.5	33.1	—	22.4	20.3	22.0	20.8
45°	20.4	35.0	22.4	—	40.5	31.0	33.2
60°	8.9	26.4	20.3	40.5	—	30.3	28.7
75°	10.3	23.0	22.0	31.0	30.3	—	36.2
90°	12.7	23.2	20.8	33.2	28.7	36.2	—

S4. Frequency support of the 90°–75° comparison

Table S4 lists every continuous eligible interval in the primary band. Endpoints are included, and the line spacing is 0.3125 Hz. Intervals containing a single line are retained as such. The 1060 lines cover 36.79% of the 2881-line band, rather than a single continuous interval.

Table S4. All eligible intervals for the primary-band 90°–75° pair.

Interval	Start (Hz)	End (Hz)	Lines
1	100.0000	100.6250	3
2	101.2500	110.3125	30
3	110.9375	111.2500	2
4	112.1875	114.3750	8
5	116.8750	117.1875	2
6	201.5625	203.4375	7
7	204.3750	207.1875	10
8	208.4375	235.6250	88
9	236.2500	239.6875	12
10	240.3125	308.4375	219
11	309.0625	484.6875	563
12	485.9375	485.9375	1
13	487.1875	487.1875	1
14	940.6250	941.5625	4
15	943.4375	945.3125	7
16	946.8750	947.1875	2
17	958.1250	959.0625	4
18	961.8750	962.5000	3
19	970.0000	970.3125	2
20	971.5625	1000.0000	92

Table S5. Frequency-bin distribution of the pair and common three-specimen masks. Bins exclude the upper endpoint except the final bin, which includes 1000 Hz.

Bin (Hz)	All lines	Pair lines	Pair coverage (%)	Common lines
100–200	320	45	14.06	45
200–300	320	308	96.25	290
300–400	320	319	99.69	39
400–500	320	274	85.62	44

Bin (Hz)	All lines	Pair lines	Pair coverage (%)	Common lines
500–600	320	0	0.00	0
600–700	320	0	0.00	0
700–800	320	0	0.00	0
800–900	320	0	0.00	0
900–1000	321	114	35.51	0

The common 0°/90°/75° mask retains 418 lines, of which 290 lie in 200–300 Hz. This concentration limits representativeness. On the identical mask, 0°–75° has 180.6% error and 90°–75° has 17.3% error; neither is an estimate of performance over excluded frequencies.

S5. Processing sensitivity and the recorded upper-frequency range

The main manuscript Table 5 gives the full minimum-output-count sweep for the 90°–75° pair. These thresholds change both the retained observations and the errors; they are deterministic processing-sensitivity analyses, not estimates of impact-to-impact variance. No individual impact records or altered-support measurements were available.

Table S6. Weak-driving-point sensitivity within 100–1000 Hz. The threshold excludes a frequency if the driving-point magnitude is less than the listed fraction of the norm of the other 14 responses. Dual applies the test to both reference and target; reference-only applies it to the reference. All rows otherwise use coherence 0.90 and at least eight outputs. No regularization is used in the baseline.

Pair	Test	Threshold (%)	Error (%)	Coverage (%)
0°–15°	Reference only	0.0	52.9	62.1
0°–15°	Reference only	1.0	52.9	62.1
0°–15°	Reference only	2.5	52.9	62.1
0°–15°	Reference only	5.0	51.0	61.3
0°–15°	Reference only	10.0	48.7	58.5
0°–15°	Dual	0.0	52.9	62.1
0°–15°	Dual	1.0	52.9	62.1
0°–15°	Dual	5.0	50.6	58.3
0°–15°	Dual	10.0	42.2	47.0
0°–30°	Reference only	0.0	149.7	65.3
0°–30°	Reference only	1.0	149.7	65.3
0°–30°	Reference only	2.5	149.7	65.3
0°–30°	Reference only	5.0	149.6	64.5
0°–30°	Reference only	10.0	148.0	60.4
0°–30°	Dual	0.0	149.7	65.3
0°–30°	Dual	1.0	149.7	65.3

Pair	Test	Threshold (%)	Error (%)	Coverage (%)
0°–30°	Dual	5.0	150.1	60.8
0°–30°	Dual	10.0	149.9	38.7
90°–75°	Reference only	0.0	18.2	36.8
90°–75°	Reference only	1.0	18.2	36.8
90°–75°	Reference only	2.5	18.2	36.8
90°–75°	Reference only	5.0	18.2	36.8
90°–75°	Reference only	10.0	18.1	36.3
90°–75°	Dual	0.0	18.2	36.8
90°–75°	Dual	1.0	18.2	36.8
90°–75°	Dual	5.0	18.2	36.8
90°–75°	Dual	10.0	17.9	32.8

The exclusion norm uses all 14 measured non-P8 mobilities before coherence masking, including low-coherence components. The test is applied in addition to the unchanged coherence/minimum-output requirements. Reference-only exclusions use the reference column and P8; dual exclusions additionally require all 14 target outputs and are retrospective. They assess numerical processing dependence, not passivity, causality, stability, statistical uncertainty, or a prospective P8-only acceptance rule.

Table S7. Representative comparisons in the original and additional frequency bands. Each band contains different samples. The comparison 60°–45° has no eligible lines at 5000–8000 Hz and is shown with an em dash rather than assigned zero error. The following paragraph summarizes the full 42-pair upper-band calculations.

Pair	Band (Hz)	Error (%)	Min. error (%)	Coverage (%)
90°–75°	100–1000	18.2	16.5	36.8
90°–75°	>1000–3000	98.4	76.2	36.2
90°–75°	>3000–5000	55.5	42.4	13.6
90°–75°	>5000–8000	131.0	91.8	4.0
0°–75°	100–1000	181.4	87.3	23.7
0°–75°	>1000–3000	153.6	91.6	10.3
0°–75°	>3000–5000	106.9	98.7	16.8
0°–75°	>5000–8000	105.9	97.5	6.4
60°–45°	100–1000	54.6	40.6	37.7
60°–45°	>1000–3000	63.8	54.5	40.5
60°–45°	>3000–5000	70.0	69.0	6.1
60°–45°	>5000–8000	—	—	0.0

Across the 42 directed CFRP comparisons, all 42 had eligible samples at 3000–5000 Hz, with errors of 55.5–566.7% and frequency coverage of 1.64–32.59%. At 5000–8000 Hz, 32 comparisons were eligible, with errors of 84.0–386.9% and coverage of 0.03–8.27%; 10 comparisons had no eligible samples. All 10 correspond to both directions of 45° with 0°, 15°, 30°, 60°, and 75°. The recalculated frequency and output masks are symmetric under reversal of every reference–target pair. Tables S8–S9 provide the complete tabulated upper-band results. Export bandwidth provenance remains unresolved (main manuscript Section 2.2).

S6. Complete upper bands and additional diagnostics

Table S8 gives the results for all 42 directed CFRP comparisons in this band. Each cell gives eligible lines / complex error (%) / minimum scalar error (%) / frequency coverage (%). A dash denotes no eligible lines, with zero coverage. The denominator is 6400 frequency lines. Values are rounded for presentation.

Table S8. All 42 CFRP comparisons within 3000 < f ≤ 5000 Hz.

Ref. / target	0°	15°	30°	45°	60°	75°	90°
0°	—	2086 / 172.7 / 90.0 / 32.59	543 / 119.1 / 95.1 / 8.48	809 / 134.6 / 96.3 / 12.64	105 / 100.2 / 97.9 / 1.64	1078 / 106.9 / 98.7 / 16.84	1071 / 112.5 / 99.2 / 16.73
15°	2086 / 115.1 / 84.8 / 32.59	—	976 / 121.0 / 98.9 / 15.25	564 / 136.4 / 95.7 / 8.81	186 / 293.2 / 89.8 / 2.91	998 / 119.2 / 97.0 / 15.59	1407 / 131.3 / 94.4 / 21.98
30°	543 / 248.7 / 92.7 / 8.48	976 / 215.2 / 88.4 / 15.25	—	469 / 283.3 / 76.1 / 7.33	229 / 386.7 / 83.8 / 3.58	623 / 147.4 / 94.2 / 9.73	644 / 164.2 / 95.5 / 10.06
45°	809 / 360.9 / 96.8 / 12.64	564 / 566.7 / 85.2 / 8.81	469 / 141.1 / 77.7 / 7.33	—	392 / 94.3 / 65.3 / 6.12	164 / 102.5 / 94.9 / 2.56	319 / 120.3 / 96.4 / 4.98
60°	105 / 431.3 / 98.5 / 1.64	186 / 98.6 / 77.7 / 2.91	229 / 121.5 / 86.1 / 3.58	392 / 70.0 / 69.0 / 6.12	—	347 / 159.6 / 82.3 / 5.42	207 / 111.7 / 84.4 / 3.23
75°	1078 / 124.1 / 93.0 / 16.84	998 / 198.1 / 92.0 / 15.59	623 / 215.8 / 94.9 / 9.73	164 / 134.6 / 92.9 / 2.56	347 / 137.6 / 80.3 / 5.42	—	871 / 132.7 / 47.8 / 13.61
90°	1071 / 218.7 / 83.8 / 16.73	1407 / 169.1 / 91.1 / 21.98	644 / 164.8 / 93.9 / 10.06	319 / 421.6 / 92.7 / 4.98	207 / 93.4 / 89.3 / 3.23	871 / 55.5 / 42.4 / 13.61	—

Table S9 gives the results for all 42 directed CFRP comparisons in this band. Each cell gives eligible lines / complex error (%) / minimum scalar error (%) / frequency coverage (%). A dash denotes no eligible lines, with zero coverage. The denominator is 9600 frequency lines. Values are rounded for presentation.

Table S9. All 42 CFRP comparisons within 5000 < f ≤ 8000 Hz.

Ref. / target	0°	15°	30°	45°	60°	75°	90°
0°	—	446 / 84.7 / 74.0 / 4.65	592 / 136.4 / 98.7 / 6.17	0 / — / — / 0.00	156 / 101.5 / 98.0 / 1.62	614 / 105.9 / 97.5 / 6.40	794 / 124.1 / 86.3 / 8.27
15°	446 / 367.7 / 74.6 / 4.65	—	292 / 212.8 / 97.4 / 3.04	0 / — / — / 0.00	3 / 386.9 / 98.1 / 0.03	109 / 204.4 / 92.9 / 1.14	429 / 257.3 / 94.4 / 4.47
30°	592 / 194.3 / 98.5 / 6.17	292 / 102.1 / 97.6 / 3.04	—	0 / — / — / 0.00	23 / 197.0 / 99.0 / 0.24	70 / 135.0 / 93.6 / 0.73	138 / 124.1 / 91.6 / 1.44
45°	0 / — / — / 0.00	0 / — / — / 0.00	0 / — / — / 0.00	—	0 / — / — / 0.00	0 / — / — / 0.00	215 / 277.4 / 92.0 / 2.24
60°	156 / 260.6 / 97.7 / 1.62	3 / 100.6 / 98.1 / 0.03	23 / 125.0 / 99.0 / 0.24	0 / — / — / 0.00	—	96 / 84.0 / 70.3 / 1.00	92 / 124.9 / 99.8 / 0.96
75°	614 / 373.1 / 97.6 / 6.40	109 / 99.7 / 92.7 / 1.14	70 / 201.8 / 94.1 / 0.73	0 / — / — / 0.00	96 / 103.4 / 69.9 / 1.00	—	388 / 121.0 / 93.6 / 4.04
90°	794 / 280.1 / 92.1 / 8.27	429 / 128.9 / 94.9 / 4.47	138 / 279.0 / 91.8 / 1.44	215 / 124.2 / 91.8 / 2.24	92 / 176.2 / 99.7 / 0.96	388 / 131.0 / 91.8 / 4.04	—

Table S10. Point weights for 90°–75° within 100–1000 Hz. Squared amplitudes are not mechanical energy.

Point	Valid cells	Local error (%)	Target squared-amplitude share (%)
1	400	43.2	0.887
2	358	18.7	0.110
3	499	17.9	14.252
4	1040	49.3	1.335
5	974	24.3	0.153
6	371	91.3	0.232
7	995	6.2	9.862
9	957	20.7	14.843
10	935	101.5	0.199
11	1042	32.1	0.127
12	824	56.2	0.416
13	353	15.3	5.775
14	1056	14.9	25.175
15	1059	12.7	26.634

Table S11. Frequency-bin weights for the same comparison. Bins exclude the upper endpoint except the last bin.

Bin (Hz)	Valid cells	Cell coverage (%)	Target squared-amplitude share (%)
100–200	525	11.72	0.714
200–300	3414	76.21	96.830
300–400	3231	72.12	1.646
400–500	2482	55.40	0.369
500–600	0	0.00	0.000
600–700	0	0.00	0.000
700–800	0	0.00	0.000
800–900	0	0.00	0.000
900–1000	1211	26.95	0.441

Table S12a. Output-count sensitivity for 0°–15° within 100–1000 Hz.

Coherence	Min. outputs	Eligible lines	Error (%)	Minimum (%)	Coverage (%)
0.9	4	1915	53.1	45.2	66.5
0.9	6	1844	53.1	45.1	64.0
0.9	8	1789	52.9	45.1	62.1
0.9	10	1549	50.3	45.0	53.8
0.9	12	1131	50.0	44.9	39.3
0.9	14	507	47.4	43.9	17.6
0.95	4	1701	48.7	43.4	59.0
0.95	6	1646	48.6	43.3	57.1
0.95	8	1518	48.1	43.2	52.7
0.95	10	1161	45.3	43.2	40.3
0.95	12	498	44.2	42.9	17.3
0.95	14	114	42.3	41.3	4.0

Table S12b. Output-count sensitivity for 0°–30° within 100–1000 Hz.

Coherence	Min. outputs	Eligible lines	Error (%)	Minimum (%)	Coverage (%)
0.9	4	2202	148.9	92.5	76.4
0.9	6	2023	149.5	92.6	70.2
0.9	8	1880	149.7	92.6	65.3
0.9	10	1358	150.1	92.6	47.1
0.9	12	708	150.7	92.3	24.6
0.9	14	279	153.7	91.2	9.7
0.95	4	1848	147.2	92.8	64.1
0.95	6	1566	147.5	92.9	54.4
0.95	8	1114	148.3	93.4	38.7
0.95	10	348	150.0	94.5	12.1
0.95	12	56	151.3	94.4	1.9
0.95	14	0	—	—	0.0

For 0°–15°, changing the output-count requirement from eight to 14 at coherence 0.90 changes error/coverage from 52.9%/62.1% to 47.4%/17.6%. For 0°–30°, the corresponding change is 149.7%/65.3% to 153.7%/9.7%. Selection therefore does not create a low-error scalar fit for the latter case. At coherence 0.95 with all 14 outputs required, 0°–30° has no eligible lines and its error is undefined, whereas 0°–15° retains 114 lines with 42.3% error and 4.0% frequency coverage (Tables S12a–S12b). An empty mask is not assigned zero error.

Table S13. Recorded or author-reported information and limits of traceability.

Item	Available basis	Unresolved information
Aligned inputs	Exact equality with all 16 original text exports after point-ID sorting	Individual impact records unavailable
Frequency and units	Numerical grid 0.3125–8000 Hz; 0.3125 Hz spacing; exported (m/s)/N and +Z labels	Original estimator and calibration/sign conversions not independently recorded
Acquisition metadata	Header Sample frequency 16000.6 Hz; Span 6250.24 Hz; Weighting Linear	Span conflicts with numerical endpoint; original acquisition project, detailed averaging and trigger settings, record duration, and independent sign/calibration conversion records were not supplied
Dimensions and fabrication	Author-reported measured ranges; common CFRP parent panel; separate matrix panel	Individual measurement sheets, instrument resolution, panel variation and detailed matrix mold/pressure records not supplied
Hammer and windows	Author-reported steel tip; software-suggested force-window cutoff of 1.12%; response exponential-window decay of 100%, equivalent to uniform weighting without exponential attenuation	Contemporaneous settings files and force-spectrum screenshots were not supplied; finite-record leakage and input-window effects were not independently quantified

Item	Available basis	Unresolved information
Support and repeats	Author-reported foam on a SUS304 surface plate supported by four air-suspension units; ten impacts per response location	Foam stiffness, damping, and contact conditions were not characterized; no altered-support test, independently tested specimen replication, or individual-impact variance estimate

Checks of the retained averaged FRF arrays gave minimum reference and target P8 magnitudes of 0.00845392 and 0.00581156 (m/s)/N, respectively, and a maximum absolute P8 ratio of 1.64061 for 90°–75° in the primary band. These descriptive values apply to the retained pairwise mask; they are not condition-number or uncertainty bounds. Table S13 distinguishes information retained in the exports from author-reported experimental details and unavailable records. Availability of additional supporting data follows Section S7.

S7. Data availability

The data presented in this study are included in the article and its Supplementary Materials. Additional supporting data, where available, may be obtained from the corresponding author upon reasonable request.

Tables S1–S13, including Tables S12a and S12b, and Figure S1 contain the supplementary numerical results and diagnostic evidence reported here. Requests for additional supporting data should identify the comparison, frequency band, or processing result of interest. Individual impact records, independently tested specimen replicates, and measurements under altered support conditions were unavailable for this study.
